# Revision of the genus *Aseptis* McDunnough (Lepidoptera, Noctuidae, Noctuinae, Xylenini) with a description of two new genera, *Paraseptis* and *Viridiseptis*

**DOI:** 10.3897/zookeys.527.9575

**Published:** 2015-10-15

**Authors:** Tomas Mustelin, Lars G. Crabo

**Affiliations:** 1 San Diego Natural History Museum, San Diego, California; 11904 Tallwood Court, Maryland 20854, USA; 2 Washington State University Adjunct Faculty; 724 14th Street, Bellingham, Washington 98225

**Keywords:** DNA barcode

## Abstract

The genus *Aseptis* McDunnough (Lepidoptera, Noctuidae, Noctuinae, Xylenini, Xylenina) is revised to include 15 species based on morphological and molecular data. Several new synonymies are introduced. In addition, two genera are described because of significant morphological differences from *Aseptis*: *Paraseptis* **gen. n.**, and *Viridiseptis* **gen. n.**, resulting in the new combinations *Paraseptis adnixa* (Grote), **comb. n.**, and *Viridiseptis marina* (Grote), **comb. n.** Although this work is primarily based on morphological data, DNA sequence data for the 658-base pair “barcode” segment of the mitochondrial gene for subunit 1 of cytochrome c oxidase was used as a secondary support for taxonomic changes within *Aseptis* and for the two new genera. Our work should provide clarity and stability in a previously difficult genus.

## Introduction

The genus *Aseptis* was described by McDunnough in 1937. A typical feature of the genus is the indentation of the outer margin of the hindwing beneath its apex between veins M1 and M3. Most species are rather dull gray or brown, many with diffuse maculation in darker brown or black. Several species display marked variation between different geographical areas, as well as within any given locality. This often correlates with the habitat and tends to yield paler and more diffusely marked individuals in dry and sandy habitats, and darker more contrasting specimens in moist and lush habitats such as in the Pacific Northwest. Many of these forms were described as separate species, resulting in many more names than true species. From a total of 31 published species names the latest checklist of North American Noctuoidea (Lafontaine and Schmidt 2010) contains 24 species, including “*Aseptis*” *marina* (Grote), which is associated tentatively with *Aseptis*. In this revision the number of species is reduced further to 17, of which15 are retained in *Aseptis* and two are placed in new genera.

Most species of *Aseptis* were described in the late 1800s and the first two decades of the 1900s, with exception of four recent ones from southern California (Mustelin et al. 2000, Mustelin 2006). At the time of these older descriptions, the West was a frontier and entomological collecting was restricted to a few localities such as Pacific ports, Provo, Utah, a few localities in Colorado, and scattered sites sampled during geological expeditions. Hence it is not surprising that short series from disparate sites were described as new species without more thorough comparisons or anatomical examination. Indeed, *Aseptis binotata* (Walker) was described seven times including in three different genera in a single publication in 1865. The material accumulated in public collections over the last 100 years now reveals that many species are polymorphic, while others are remarkably constant.

The combination of too many names, geographical variation, and the fact that many *Aseptis* are similar gray-brown moths with diffuse markings has given rise to considerable confusion in public collections. A lack of published illustrations of most species since Barnes & McDunnough’s publications a century ago (Barnes and McDunnough 1912a) has also contributed to a lack of clarity. In this revision, we define the status of the species of *Aseptis*, describe two new genera for species previously associated with the genus, and illustrate one or more representative adult specimens and the genitalia of all of them.

## Materials and methods

**Figures 1–2.**
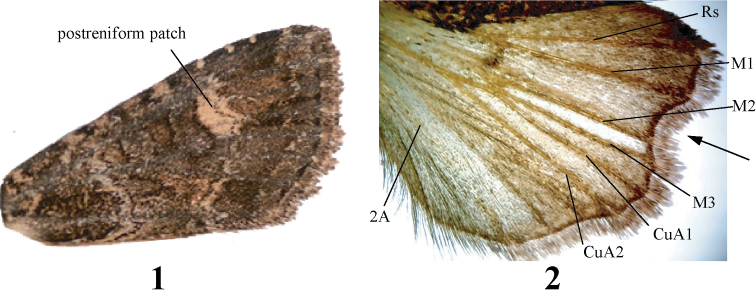
Characters and nomenclature of the genus *Aseptis*. **1** Forewing of *Aseptis binotata* **2** hindwing of male *Aseptis fumosa*. The arrow points at the indentation typical of *Aseptis*.

Wing pattern and genitalia structure terminology follow Lafontaine (2004). Terms not defined in this reference are the penicillus, a broad-based lateral extension of the tegumen near the attachment to the vinculum (Forbes 1954), and the term “postreniform patch” which we introduce for a pale area abutting the lateral reniform spot in the distal medial and adjacent postmedial areas of the forewing (Fig. 1).

The male and female genitalia were prepared using standard methods (Hardwick 1950, Lafontaine 2004). Briefly, the detached abdomen was soaked in 10% KOH to dissolve soft tissues. Dissection was performed initially in water followed by hardening with isopropyl alcohol or ethanol. The male vesica and female bursa were inflated. The preparations were stained with Chlorazole Black or orcein and were mounted in Euparal on glass slides.

The 658 base pair DNA “barcode” region of the mitochondrial cytochrome *c* oxidase subunit 1 (CO1) was used to assess molecular variation of the species included in *Aseptis* and related genera in recent check lists. Previously submitted samples available as of February, 2015 at the Barcodes of Life Campaign (BOLD) at the University of Guelph (Ontario, Canada) had been analyzed by standard DNA extraction, amplification, and sequencing protocols for the BOLD initiative as described by Hebert et al. (2003). The barcode sequences were compared using similarity trees obtained using the Kimura-2-Parameter distance model as implemented on the Barcode of Life Data Systems website (http://www.barcodinglife.org). Table 1 lists the major haplotypes of specimens examined in this study.

**Table 1.** 

Species	Haplotype	Voucher #	Seq. length	Country	State/Prov.	Exact Site	Lat	Lon	Collectors	Deposition
*Aseptis binotata*	ABI1	CNCNoctuoidea12188	658[0n]	USA	CA	Pine Mountain, Ventura Co.			T. Dimmock	CNC
*Aseptis binotata*	ABI2	CNCNoctuoidea12190	658[0n]	USA	CA	Laguna Mountains, San Diego Co.			T. Mustelin	CNC
*Aseptis binotata* (*bultata*)	ABI3	CNCNoctuoidea12200	658[0n]	USA	UT	Capital Reef, Garfield Co.			P. Opler	CNC
*Aseptis binotata* (*dilara*)	ABI4	TMustelin#319	609[0n]	USA	CO	John Brown Canyon, Mesa Co			J.S. Nordin	TMC
*Aseptis binotata* (*genitrix*)	ABI5	CNCNoctuoidea12164	658[0n]	USA	WY	Upper Blair P.G. north of Rd 705, Albany Co.			J.S. Nordin	CNC
*Aseptis binotata* (*genitrix*)	ABI6	CNCNoctuoidea12165	658[0n]	USA	NV	11 mi SW Wells			Lafontaine and Troubridge	CNC
*Aseptis binotata* (*genitrix*)	ABI7	TMustelin#317	658[0n]	USA	WY	Fox Creek, Albany Co			J.S. Nordin	TMC
*Aseptis catalina*	ACA1	CNCNoctuoidea12193	658[0n]	USA	CA	Anza Borrego, San Diego Co.			T. Mustelin	CNC
*Aseptis catalina*	ACA2	CNCNoctuoidea12196	658[0n]	USA	CA	Anza Borrego, San Diego Co.			T. Mustelin	CNC
*Aseptis catalina*	ACA3	CNCNoctuoidea12197	658[0n]	USA	AZ	Hwy 88 12miNE Apache Jct, Maricopa Co.			J. Troubridge	CNC
*Aseptis characta*	ACH1	CNCNoctuoidea12207	658[0n]	USA	WA	Bridgeport	48	-119,617	J. Troubridge	CNC
*Aseptis characta*	ACH2	CNCNoctuoidea12209	658[0n]	Canada	BC	Mount Kobau	49,1	-119,65	J. Troubridge	CNC
*Aseptis characta*	ACH3	CNCNoctuoidea12212	658[0n]	USA	NV	Angel Lake	41,01	-115,04	Troubridge and Lafontaine	CNC
*Aseptis characta*	ACH4	CNCNoctuoidea13376	658[0n]	USA	CA	San Bernardino Mountains, San Bernardino Co.	34,175	-116,803	T&S Mustelin	CNC
*Aseptis ethnica*	AET1	CNCNoctuoidea12175	658[0n]	USA	CA	Magalia, Butte Co.			L. Crabtree	CNC
*Aseptis ethnica*	AET2	TMustelin#193	592[0n]	USA	CA	2 mi south of Lake Henshaw, San Diego Co			T. Mustelin	TMC
*Aseptis ethnica*	AET3	TMustelin#258	609[0n]	USA	OR	Illinois River, Josephine Co	42,75	-123,683	J. Troubridge	TMC
*Aseptis fanatica*	AFA1	CNCNoctuoidea13155	658[0n]	USA	CA	Laguna Mountains, San Diego Co.			T.&S. Mustelin	CNC
*Aseptis fanatica*	AFA2	CNCNoctuoidea13378	658[0n]	USA	CA	Laguna Mountains, San Diego Co.			T. Mustelin	CNC
*Aseptis ferruginea*	AFE	CNCNoctuoidea12170	658[0n]	USA	CA	2miNE of Julian, San Diego Co.			T. Mustelin	CNC
*Aseptis fumeola*	AFUE1	CNCNoctuoidea12176	658[0n]	USA	CA	Laguna Mountains, San Diego Co.			T. Mustelin	CNC
*Aseptis fumeola*	AFUE2	CNCNoctuoidea13380	658[0n]	USA	CA	Laguna Mountains, San Diego Co.			T. Mustelin	CNC
*Aseptis fumosa*	AFUM	CNCNoctuoidea12161	658[0n]	USA	CA	Pine Mountain, Ventura Co.			T. Dimock	CNC
*Aseptis lichena*	ALI	TMustelin#318	609[0n]	USA	CA	Twain Harte, [? County],			Lundgren	TMC
*Aseptis murina*	MU	CNCNoctuoidea12173	612[0n]	USA	CA	Inaja Picnic Ground, San Diego Co.			T. Mustelin	CNC
*Aseptis perfumosa*	APE1	CNCNoctuoidea12127	658[0n]	USA	CA	Laguna Mountains, San Diego Co.			T. Mustelin	CNC
*Aseptis perfumosa*	APE2	CNCNoctuoidea12129	658[0n]	USA	CA	Peñasquitos Canyon, San Diego			T. Mustelin	CNC
*Aseptis perfumosa*	APE3	TMustelin#307	609[0n]	USA	CA	Upper Ojai Valley, Ventura Co			T.E. Dimock	TMC
*Aseptis pseudolichena*	APS	CNCNoctuoidea12148	658[0n]	USA	CA	Laguna Mountains, San Diego Co.			T. Mustelin	CNC
*Aseptis serrula*	ASE	TMustelin#316	572[0n]	USA	CA	In-Ko-Pah Gorge, Imperial Co			T. Mustelin and N. Bloomfield	TMC
*Aseptis susquesa*	ASU1	TMustelin#22	603[0n]	USA	CA	San Diego				TMC
*Aseptis susquesa* (*monica*)	ASU2	TMustelin#15	609[0n]	USA	CA	Scissors Crossing, San Diego Co.			T. Mustelin	TMC
*Aseptis susquesa* (*monica*)	ASU3	TMustelin#321	609[0n]	USA	CA	Laguna Mountains, San Diego Co.			T. Mustelin	TMC
*Aseptis torreyana*	ATO	TMustelin#284	609[0n]	USA	CA	Torrey Pines State Reserve, San Diego			N. Bloomfield	TMC
*Paraseptis adnixa*	PAD1	CNCNoctuoidea12201	658[0n]	Canada	BC	Vancouver Island, Saanichton			J. Troubridge	CNC
*Paraseptis adnixa*	PAD2	CNCNoctuoidea12202	658[0n]	USA	CA	Lee Vining, Mono Co.	37,941	-119,123	J. Troubridge, L. Crabo	CNC
*Paraseptis adnixa*	PAD3	CNCNoctuoidea12206	658[0n]	Canada	BC	Kirby Flats			J. Troubridge	CNC
*Paraseptis adnixa* (*pausis*)	PAD4	CNCNoctuoidea12203	658[0n]	USA	CA	Laguna Mts, San Diego Co.			T. Mustelin	CNC
*Paraseptis adnixa* (*pausis*)	PAD5	CNCNoctuoidea12204	658[0n]	USA	CA	Alamo Mountain, Ventura Co.			T. Dimmock	CNC
*Paraseptis adnixa* (*pausis*)	PAD6	LEP038031	658[0n]	USA	CA	2 mi. E. Bassetts, Hy 49, Sierra Co.			P. A. & E. Opler	CNC
*Viridiseptis marina*	VMA	CNCNoctuoidea12235	658[0n]	USA	CA	McCain Valley, San Diego Co.	32,703	-116,265	T. Mustelin	CNC

This study is based on examination of material, including type specimens and genitalia preparations, in the following collections:

AMNH The American Museum of Natural History, New York, New York, USA

BMNH The Natural History Museum [British Museum of Natural History], London, UK

CNC Canadian National Collection of Insects, Arachnids, and Nematodes, Ottawa, Ontario, Canada

FMNH Field Museum of Natural History, Chicago, Illinois, USA

LACM Los Angeles County Museum, Los Angeles, California, USA

LGC Lars Crabo Collection, Bellingham, Washington, USA

MSU Michigan State University, East Lansing, Michigan, USA

ODAC Oregon Department of Agriculture, Salem, Oregon, USA

OSAC Oregon State Arthropod Collection, Corvallis, Oregon, USA

RHLC Ronald H. Leuschner Collection, now at the McGuire Center, Gainesville, Florida, USA

SDNHM San Diego Natural History Museum, San Diego, California, USA

TEDC Thomas E. Dimock Collection, Ventura, California, USA

TMC Tomas Mustelin Collection, Potomac, Maryland, USA

UCR University of California at Riverside, Riverside, California, USA

USNM National Museum of Natural History [formerly United States National Museum], Washington, District of Columbia, USA

WFBM W. F. Barr Entomological Collection, University of Idaho, Moscow, Idaho, USA

WSUC James Entomological Collection, Washington State University, Pullman, Washington, USA

ZMH Zoological Museum, Helsinki, Finland

## Results

### Key to genera included in *Aseptis* McDunnough sensu Lafontaine & Schmidt, 2010

**Table:** 

1	Male vesica with apical long spine-like cornutus; posterior half of female ductus bursae membranous	***Aseptis***
–	Male vesica apex lacking cornutus or with minute cornutus; posterior half of female ductus bursae at least partially sclerotized	**2**
2	Ampulla of male clasper present; female corpus bursae with four long signa and posterior ductus bursae sclerotized circumferentially	***Paraseptis***
–	Ampulla of male clasper absent; female corpus bursae lacking signa and posterior ductus bursae with sclerotized plate in ventral wall	***Viridiseptis***

### Key to the genus *Aseptis* McDunnough

**Table:** 

1	Male	**2**
–	Female	**19**
2	Antenna serrate	***Aseptis serrula***
–	Antenna filiform	**3**
3	Digitus absent	**4**
–	Digitus elongate, narrow	**8**
4	Ventral cucullus with spike-like process; forewing mottled olive green to olive yellow; California	**5**
–	Ventral cucullus normal, rounded; forewing not olive; widespread, including California	**6**
5	Forewing ground color olive green with yellow tan and black pattern; Kern and Tuolumne counties, California, and north; male valve nearly straight; female genitalia indistinguishable from *Aseptis pseudolichena*	***Aseptis lichena***
–	Forewing ground color light olive to olive-yellow; Kern and Tuolumne counties, California and south; male valve bent slightly ventrad at mid-point; female genitalia indistinguishable from *Aseptis lichena*	***Aseptis pseudolichena***
6	Vesica with single cornutus; forewing mottled light yellow tan and gray; deserts of southern California and Arizona	***Aseptis catalina***
–	Vesica with two or more cornuti; forewing not as above; widespread in western North America	**7**
7	Valve slightly S-shaped; forewing mottled gray, or gray and tan	***Aseptis characta***
–	Valve nearly straight; forewing dark, blackish	***Aseptis fumosa***
8	Digitus perpendicular to valve; southern California	**9**
–	Digitus oblique to valve, pointed ~45° toward ventral cucullus; widespread, including southern California	**10**
9	Digitus origin near ventral valve with most of it below ventral valve margin; forewing dark brown to black brown; widespread in southern California	***Aseptis perfumosa***
–	Digitus origin on mid-valve near base of ampulla, barely reaching ventral margin; immediate coast near San Diego, California	***Aseptis torreyana***
10	Aedeagus longer, > 4× as wide as long; smaller narrower-winged species (wingspan ≤ 35 mm); forewing with contrasting light postreniform patch or small black basal dash	**11**
–	Aedeagus stout, ≤ 4× as wide as long; large broad-winged species (wingspan ≥ 35 mm); forewing without basal dash and usually without postreniform patch	***Aseptis fumeola* species group**	**12**
11	Small basal dash present, evident in all but the darkest specimens; forewing a shade of brown; hindwing base gray; widespread in western North America	***Aseptis binotata***
–	Basal dash absent; forewing gray with patches of pale rusty brown; hindwing base white with streaks extending distally; deserts of Southwest and southern California	***Aseptis susquesa***
12	Forewing smooth gray with pale costa; maculation reduced to dark filling of spots and dotted lines; extreme southern California	***Aseptis murina***
–	Forewing not as above, costa similar to rest of wing; West Coast and parts of Southwest, including southern California	**13**
13	Forewing mottled gray brown with conspicuous gray filling of spots, small yellowish postreniform spot, and irregular black to dark gray shade proximal to subterminal line; California, Arizona, and southern Utah and Nevada	***Aseptis fumeola***
–	Forewing light or dark but more uniform, filling of spots not strongly contrasting, medial area between reniform spot and postmedial line not significantly lighter than rest of wing or reddish in central and northern California; California, Arizona, Oregon, and Washington	**14**
14	Forewing ground color strongly red brown	**15**
–	Forewing not red brown, sometimes patchy reddish areas near reniform spot in central and northern California	**18**
15	Forewing rusty red brown with darker markings; extreme southern California	***Aseptis ferruginea***
–	Forewing bright red brown, occasionally with darker markings; San Benito County, California	***Aseptis fanatica* (part)**
16	Southern California	**17**
–	Central California to Washington	**18**
17	Forewing ground color slightly mottled pale gray brown; male valves oriented ≥ 120° relative to each when mounted flat; female corpus bursae ~5× as long as wide	***Aseptis ethnica* (part)**
–	Forewing ground color medium to dark brown; male valves oriented at ~90° when displayed similarly; female corpus bursae ~7× as long as wide	***Aseptis fanatica* (part)**
18	Forewing ground color slightly mottled brown, usually with evident dark shade preceding subterminal line and often with reddish postreniform spot; male valves oriented ≥ 120° relative to each when mounted flat; female corpus bursae ~5× as long as wide	***Aseptis ethnica* (part)**
–	Forewing ground color blackish brown, maculation faint; male valves oriented at ~90° when displayed similarly; female corpus bursae ~7× as long as wide	***Aseptis fanatica* (part)**
19	Corpus bursae elongate, 5–7× as long as wide, with small cone-shaped appendix bursae; papilla analis without long hair-like basal seta	***Aseptis fumeola* species group**... **12**
–	Corpus bursae wider, ovoid, 1.3–1.5× as long as wide, with appendix bursae not as above; papilla analis with sparse or thick hair-like basal setae	**20**
20	Forewing mottled olive green to yellow green; California	**5**
–	Forewing ground color gray or brown; widespread, including California	**21**
21	Apex of papilla analis with a thin sclerotized flange	***Aseptis perfumosa***
–	Apex of papilla analis lacking a flange	**22**
22	Hairs at base of papilla analis dense, with expanded ventral patches; Southwest deserts and southern California	**23**
–	Papilla analis with a sparse single row of basal hair-like setae; widespread, including southern California	**24**
23	Papilla analis covered with short needle-like setae, medial dorsal margin smooth; forewing patchy light yellow tan and gray	***Aseptis catalina***
–	Papilla analis rugose, scale-like, dorsal medial margin irregular; forewing gray with few light marks	***Aseptis serrula***
24	Ground color of forewing dark smoky brown to nearly black; appendix bursae bluntly rounded and mediolaterally compressed	***Aseptis fumosa***
–	Ground color variable, brown to gray; if dark brown then more light scaling including filling of lines and ochre postreniform patch; appendix bursae not as above, asymmetric	**25**
25	Ground color of forewing a shade of brown (including gray brown), but lacking extensive gray areas	***Aseptis binotata***
–	Forewing gray or gray with light tan or rusty accents	**26**
26	Forewing mottled light gray, with at most minor patches of olive or tan scales	**27**
–	Forewing gray with extensive tan or rusty-tan scales	**28**
27	Forewing mottled light gray, markings include a thin black basal dash; Pacific Coast near San Diego, California; female genitalia unknown	***Aseptis torreyana***
–	Forewing mottled light and medium dark gray to darker medium gray, black dash absent; widespread, including in southern California; appendix bursae broad based but short; corpus bursae lacking signa	***Aseptis characta* (part)**
28	Forewing pattern longitudinally streaked, with rusty patches that are most prominent in fold and distal to reniform spot; bursa copulatrix with corpus bursae and appendix bursae nearly equal in size; desert Southwest and southern California	***Aseptis susquesa***
–	Forewing pattern mottled gray and tan or rusty tan, not streaky; appendix bursae much smaller than corpus bursae; widespread in western North America	***Aseptis characta* (part)**

#### Aseptis

Taxon classificationAnimaliaLepidopteraNoctuidae

McDunnough, 1937

Aseptis McDunnough, 1937: 59.

##### Type species.

*Hadena genitrix* Grote (a synonym of *Aseptis binotata* (Walker)) by original designation.

##### Diagnosis.

*Aseptis* is a moderate-sized genus of medium-sized noctuids (wingspan 27.5–45.0 mm) in the subtribe Xylenina Guenée of the tribe Xylenini Guenée of the subfamily Noctuinae Latreille (Lafontaine and Schmidt 2010) from western North America. Adults are typically dull mottled gray or brown, although a few species are red brown or nearly black, with typical noctuid lines and spots, often with a pale patch in the medial and postmedial areas abutting the lateral reniform spot (“postreniform patch” (Fig. 1)), which is easily mistaken for the reniform spot. The reniform, orbicular, and claviform spots are present in most species and are often large and closely positioned; the reniform spot is usually figure-eight shaped. The hindwing outer margin is concave between M1 and M3, M2 is visible and is closer to M3 than M1, and the wing is often palest with loss of scales between M1 and M3 (Fig. 2). The male antenna is filiform, serrate in *Aseptis serrula* (Barnes & McDunnough). The male abdomen has basal coremata with pockets extending on segments one and two. The male genitalia have a narrow sharply-pointed uncus; a tegumen that is laterally compressed near the uncus base and has broad penicillus lobes; a strap-like valve with small sacculus and weakly differentiated rounded cucullus with a weak corona (ventral cucullus pointed in the *Aseptis lichena* species group), a curved ampulla of the clasper oriented perpendicular to or parallel to the costa, and an elongate triangular or spike-like digitus arising from a weakly sclerotized plate on the mid-valve (digitus absent in several species); the aedeagus is tubular with slight ventrad bend distally, and the vesica is 1¼–2 × aedeagus length with 90–180° bend ventrad at the base, and bears a long proximally-directed apical cornutus and additional 0–2 smaller cornuti and 2–3 broad-based diverticula. In the female, the papilla analis is lightly sclerotized, triangular, with a rounded tip, and is covered with short spike-like setae (rugose scales in *Aseptis serrula*) and from zero to innumerable hair-like basal setae; the ductus bursae is membranous except near the corpus bursae; the moderately-sclerotized appendix bursae is sack-like or weakly bilobed and extends posteriorly from the left ventral corpus bursae; the corpus bursae is ovoid, 1.3–7 × as long as wide, with 0–4 long narrow signa.

*Aseptis* can be distinguished from all genera other than *Paraseptis* Mustelin & Crabo and *Viridiseptis* Mustelin & Crabo, both described below, by the concave hindwing notch. *Aseptis* males have long apical cornutus on the vesica, absent or very small in the other genera. *Aseptis* females lack sclerotization of the posterior ductus bursae.

##### Distribution and biology.

*Aseptis* species mainly occur west of the Great Plains from south-central Alberta and southern British Columbia to northwestern Mexico; one species, *Aseptis characta*, extends eastward into the Great Plains as far as Manitoba. The greatest concentration of species is near the Pacific Coast, particularly in southern California, and in the desert Southwest. The adult flight season is from late spring to late summer and is often fairly long, but species in desert habitats usually fly only during the spring. They occur in a variety of habitats from forest, shrub steppe and chaparral, to desert. As typical of the tribe Xylenini (Fibiger and Lafontaine 2005), the larvae feed on the leaves of woody plants.

##### Discussion.

Fifteen *Aseptis* species are recognized herein. Seven sort easily into species groups, five in the *Aseptis fumeola* species group and two in the *Aseptis lichena* species group. No natural groupings were found for the other species.

Two species previously associated with *Aseptis* differ significantly in structure from the other members of the genus. “*Aseptis*” *marina* was moved recently to *Aseptis* from *Oligia* Hübner in the Apameini (Lafontaine and Schmidt 2010). They noted that although it appears be related to *Aseptis* they are not congeneric, adding the quotations to denote the tentative association. Its hindwing margin has a slightly concave segment like those of *Aseptis*, but the male and female genitalia are strikingly different. We describe *Viridiseptis* for it below and introduce *Viridiseptis marina* (Grote), comb. n. Its relationship to the Xylenini is also discussed further. The other outlier, *Paraseptis adnixa* (Grote), comb. n., is surprising because it superficially resembles *Aseptis binotata* but differs from *Aseptis* in several features of the male and female genitalia. In addition to the anatomic differences, the CO1 barcodes of *Viridiseptis marina* and *Paraseptis adnixa* variably sort away from *Aseptis* and each other within a large selection of Xylenini.

The name *Aseptis* was presumably chosen by James McDunnough to denote its distinction from *Septis* Hübner, a synonym of *Apamea* Ochsenheimer, in the Apameini. *Aseptis* and *Apamea* are not related closely.

### *Aseptis fumeola* species group

The *Aseptis fumeola* species group consists of five species (*Aseptis fumeola* (Hampson), *Aseptis ethnica* (Smith), *Aseptis murina* Mustelin, *Aseptis ferruginea* Mustelin, and *Aseptis fanatica* Mustelin). Its members are relatively large (wingspan ≥ 35 mm) with broad forewings with diffuse markings. The hindwing is dark, which obscures the veins. The male valve is long and narrow with a truncated or foot-shaped cucullus, upright ampulla, and oblique pointed digitus. The aedeagus is stubby, usually 3–4× as long as wide, with a large sack-like vesica with large diverticula and long apical cornutus. Females have bursae with an elongate corpus bursae and small appendix bursae, and lack basal hairs on the papillae anales. The genitalia are similar within the group and the species are most easily identified by their habitus.

*Aseptis ethnica* and *Aseptis fanatica* display geographical variation in that both are much darker at the northern than at the southern ends of their ranges. Nevertheless, members of this species group tend to be relatively constant in appearance at any given location. An exception to this is a population of *Aseptis fanatica* from San Benito County, California, in which approximately half of the specimens are bright reddish whereas the other half are of the typical black color.

All species in the *Aseptis fumeola* species group occur in California. The ranges of two of the species extend north to the Pacific Northwest, two as far East as Arizona, and two are restricted to southern California. All five species are sympatric in southern California.

The larvae of *Aseptis fumeola*, *Aseptis ethnica*, and *Aseptis fanatica* feed on Manzanita (*Arctostaphylos* spp., Ericaceae) and related plants.

The CO1 barcodes of the species in this species group are relatively similar and cluster tightly within the genus. The largest difference in barcode sequences between two species in the group is 2.4%. *Aseptis fanatica* is the only species in the species group with more than a single barcode haplotype.

#### Aseptis fumeola

Taxon classificationAnimaliaLepidopteraNoctuidae

(Hampson, 1908)

**Figures 3–18.**
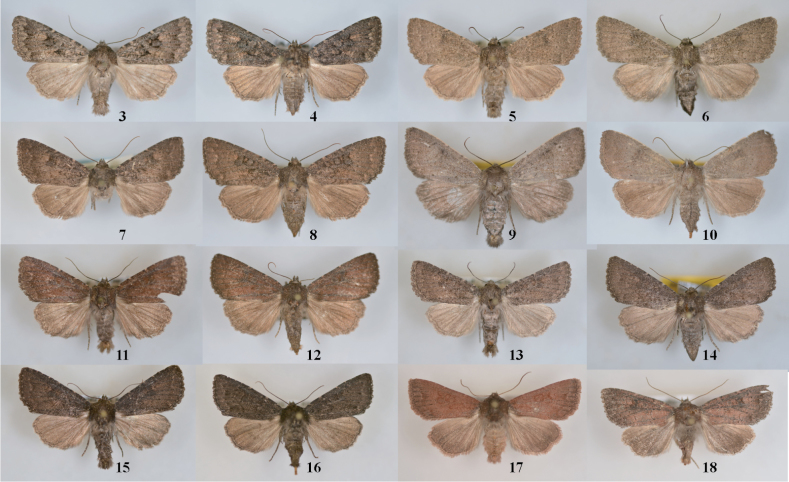
*Aseptis* adults. Aseptis fumeola species group. **3** *Aseptis fumeola*, male (San Diego Co., CA) **4** *Aseptis fumeola*, female (San Diego Co., CA) **5** *Aseptis ethnica*, male (Ventura Co., CA) **6** *Aseptis ethnica*, female, (Ventura Co., CA) **7** *Aseptis ethnica*, male (Josephine Co., OR) **8** *Aseptis ethnica*, female (Josephine Co., OR) **9** *Aseptis murina*, male Paratype (San Diego Co., CA) **10** *Aseptis murina*, female Paratype (San Diego Co., CA) **11** *Aseptis ferruginea*, male (Ventura Co., CA) **12** *Aseptis ferruginea*, female (San Diego Co., CA) **13** *Aseptis fanatica*, male Paratype (San Diego Co., CA) **14** *Aseptis fanatica*, female Paratype (San Diego Co., CA) **15** *Aseptis fanatica*, male (Kittitas Co., WA) **16** *Aseptis fanatica*, female (Kittitas Co., WA) **17** *Aseptis fanatica*, male (San Benito Co., CA) **18** *Aseptis fanatica*, female (San Benito Co., CA).

Figs 3 , 4 , 59 , 76

Trachea fumeola Hampson, 1908: 186.
Trachea (Hadena) probata Barnes & McDunnough, 1910: 153.

##### Type material.

*Trachea fumeola*: **Holotype** male [BMNH, photograph examined]. Type Locality: Pinal Mountains, Arizona. Trachea (Hadena) probata: **Holotype** female [USNM, photograph examined]. Type Locality: Huachuca Mountains, Arizona.

##### Diagnosis.

A large and broad-winged *Aseptis*, wingspan 41.1±1.6 mm (n=25; range 38.5–45.0 mm), with a dark slightly shiny gray-brown forewing with contrasting dark patches, particularly in the basal and postmedial areas. The medial area typically is paler with reddish tan near the conspicuous large black reniform spot, small round orbicular spot, and short claviform spot. The reddish postreniform patch is relatively prominent for the species group. The postmedial line usually is well marked and curves around the reniform spot. The serrated subterminal line is a prominent border between the postmedial and paler subterminal areas. The hindwing is smoky gray brown, darker in females.

*Aseptis fumeola* can be identified by its large size and patchy forewing maculation. Some northern California *Aseptis ethnica* have a similar brown forewing with reddish tan around the spots; *Aseptis fumeola* tends to be more contrasting, less reddish, and a bit larger. Occasional *Aseptis perfumosa* specimens are dark brown with reddish suffusion in the upper medial area. This form is always much smaller and darker than *Aseptis fumeola*, has a narrower forewing, and much different genitalia. The male and female genitalia are as in the description to the species group. The male valve has a foot-shaped cucullus.

##### Distribution and biology.

*Aseptis fumeola* is known from Arizona, southern and central California, southern Nevada, and south-eastern Utah. It flies in the foothills and mountains in dry chaparral, parkland, and conifer forest. Most records are from June and July. The larva and pupa were described and figured by Comstock (1940a). The pale-green larvae were found and reared on new leaves of manzanitas (*Arctostaphylos* spp.). Crumb (1956) found it on *Aseptis pungens* Kunth in Arizona and described the larva as strange and sluggish.

##### Remarks.

The Latin name *fumeola* means smoked, likely to denote its black-peppered maculation.

#### Aseptis ethnica

Taxon classificationAnimaliaLepidopteraNoctuidae

(Smith, 1899)

Figs 5–8 , 60 , 77

Hadena ethnica Smith, 1899: 263.

##### Type material.

**Holotype** male [USNM, examined]. Type Locality: Yosemite, California.

##### Diagnosis.

*Aseptis ethnica* is a large and broad-winged, wingspan 41.2±1.5 mm (n=25; range 38.0–43.5 mm) with ill-defined markings. In southern California its forewing is dull grayish tan with a grainy appearance (Figs 5 and 6). In central and northern California and Oregon, it is dull deeper brown, sometimes with some reddish tones surrounding the dark-filled reniform and orbicular spots (Figs 7 and 8). The holotype from Yosemite is of the darker form and is reminiscent of *Aseptis fumeola*. Most *Aseptis ethnica* are much less contrasting than *Aseptis fumeola* and lack its contrasting black-outlined spots, patchy dark shading, and reddish postreniform patch. *Aseptis ethnica* can be challenging to distinguish from *Aseptis fanatica*, particularly when worn. Typically, *Aseptis ethnica* is larger with a broader forewing, has a grainier forewing pattern, and is the lighter species in any location. In southern California, *Aseptis ethnica* is pale grayish tan whereas *Aseptis fanatica* is darker gray brown. In northern California, *Aseptis ethnica* is as dark as *Aseptis fanatica* in San Diego County, but in this area *Aseptis fanatica* is nearly black. In San Diego County, *Aseptis ethnica* can be distinguished from *Aseptis murina* by being grainier and browner, and by lacking its characteristic pale costa.

The male genitalia of *Aseptis ethnica* can be distinguished from those of *Aseptis fanatica* by the angle subtended by the two valves as noted in the key. The female bursa is slightly shorter in *Aseptis ethnica* than in *Aseptis fanatica*, approximately 5× its width in *Aseptis ethnica* and 7× in the latter species.

The CO1 barcode DNA sequence of *Aseptis ethnica* is closest to *Aseptis murina*, differing by 1.8%.

##### Distribution and biology.

*Aseptis ethnica* is known from Arizona, California, western Oregon, and Baja California Norte, Mexico. The northern limit is not known precisely due to similarity of this species and *Aseptis fanatica* in this portion of its range; however, it occurs at least to Jefferson County, Oregon, based on DNA. *Aseptis ethnica* flies in open Pine and Oak forest and mountain chaparral, mostly at elevations of above 1500 m in southern California but at lower elevations farther north. It has been found from early May to August and can be locally abundant during its peak flight in June and July. In the mountains of southern California (e.g., Laguna, Volcan, Palomar, and Santa Rosa Mountains) it often flies together with *Aseptis fumeola*, *Aseptis fanatica*, and occasional specimens of *Aseptis ferruginea*. The immature stages are unknown, but the larval food plant was revealed by J.B. Smith’s description of the species in 1899 based on adult specimens raised from caterpillar on manzanitas (*Arctostaphylos* spp.).

##### Discussion.

The name *ethnica* is Latin and means heathen. Perhaps Smith was inspired by the raw wilderness of Yosemite in late 1899.

#### Aseptis murina

Taxon classificationAnimaliaLepidopteraNoctuidae

Mustelin, 2000

Figs 9 , 10 , 61 , 78

Aseptis murina Mustelin, 2000. In: Mustelin et al. 2000: 8.

##### Type material.

**Holotype** male [SDNHM, examined]. Type locality: Inaja Picnic Ground, San Diego County, California.

##### Diagnosis.

This is a large species with perhaps the broadest forewing in the group, wingspan 40.0±1.0 mm (n=12; range 39–42 mm). The forewing is smooth gray with a slightly bluish sheen when fresh, a diffuse and faint dark reniform spot, postmedial line of black dots, and a pale-cream costa in fresh specimens. The hindwing is barely a shade paler than the forewing.

This species can be identified by the combination of large size, smooth gray forewing with pale costa, and southern California distribution. It is most likely to be confused with *Aseptis ethnica*, especially when worn.

##### Distribution and biology.

*Aseptis murina* is known only from southern California where it occurs in coastal chaparral, foothills, mountain brush land and oak forest, and in the mountain-desert transition zone from sea level to 2000 m. It can be found with all other species of the species group. Localities for it include the Cuyamaca, Laguna, and San Gabriel Mountains, Pinyon Crest, and San Marcus Pass in Santa Barbara County. The flight period is from early May to July. The early stages are unknown.

##### Discussion.

The name *murina* is Latin and means mouse-like to denote the smooth murine appearance of the moth.

#### Aseptis ferruginea

Taxon classificationAnimaliaLepidopteraNoctuidae

Mustelin, 2000

Figs 11 , 12 , 62 , 79

Aseptis ferruginea Mustelin, 2000. In: Mustelin et al. 2000: 8.

##### Type material.

**Holotype male** [SDNHM, examined]. Type locality: Wynola, San Diego County, California.

##### Diagnosis.

*Aseptis ferruginea* is one of the smallest and most narrow-winged species in the group, wingspan 36.4±0.5 mm (n=6; range 35.5–37.0 mm). Its brown forewing is distinctly reddish, deep claret when fresh and rustier when worn, and the veins are usually black. Most specimens have a clearly-marked dark-filled reniform spot and a jagged pale subterminal line. The hindwing is distinctly paler than the forewing. The male valve differs from others in the species group in being even and straight with a perpendicular lateral cucullus that is straight or slightly concave.

*Aseptis ferruginea* can usually be identified by superficial appearance, especially its red color, and males can be confirmed by dissection. Some central California *Aseptis fanatica* are bright red brown and could be confused with it, although this morph is not known from within its geographical range; these *Aseptis fanatica* are smoother than *Aseptis ferruginea* and lack the other forewing markings described above.

The CO1 barcode sequence of *Aseptis ferruginea* is the most unique of any member of the species group. It is closest to that of *Aseptis ethnica* from which it differs by at least 2.2%.

##### Distribution and biology.

This species is endemic to southern California. All records are from San Diego County from an area between Boulevard-Manzanita near the Mexican border north to Lake Henshaw at altitudes of 800–1600 m. It flies in open oak forest, foothill chaparral, and in the mountain-desert transition zone. *Aseptis ferruginea* can be encountered together with the much more abundant *Aseptis ethnica* and *Aseptis fanatica* at higher altitudes and with *Aseptis murina* at lower elevations. Records are from late June to August. The early stages are unknown.

##### Discussion.

The name *ferruginea* is Latin and means rusty.

#### Aseptis fanatica

Taxon classificationAnimaliaLepidopteraNoctuidae

Mustelin, 2006

Figs 13–18 , 63 , 80

Aseptis fanatica Mustelin, 2006: 27.

##### Type material.

**Holotype** male [SDNHM, examined]. Type locality: Pine Cove, San Jacinto Mountains, Riverside County, California.

##### Diagnosis.

This species is similar in size and shape to *Aseptis ferruginea*, wingspan 38.9±1.6 mm (n = 25; range 35–42 mm). In southern California *Aseptis fanatica* has a dark chocolate-brown forewing (Figs 13 and 14), whereas in northern California, Oregon, and Washington it is darker brown to nearly black (Figs 15 and 16). Some individuals in central California are smooth bright red brown (Figs 17 and 18). The maculation is dark, either diffuse or weakly contrasting. The most prominent markings are the black-filled reniform spot, a black shade proximal to the incomplete pale subterminal line, and pale-yellowish spots on the costa at the antemedial and postmedial lines. Well-marked specimens have a serrate black postmedial line, some black on the veins, and scattered pale scales giving them a peppered look. The hindwing is slightly paler than the forewing, particularly in males.

Separating *Aseptis fanatica* from *Aseptis ethnica* can be challenging. As a rule, *Aseptis fanatica* is the darker species at any location. In southern California *Aseptis fanatica* is dark gray brown whereas *Aseptis ethnica* is pale tan gray. In northern California where *Aseptis ethnica* is darker and often has some reddish brown around the spots, *Aseptis fanatica* is nearly black. *Aseptis fanatica* tends to be narrower winged and smaller, but there is overlap in size. If necessary, the genital characters of the male valves and female bursae given in the key to species can be used to distinguish the two species.

*Aseptis fanatica* is the only species in the species group with two CO1 barcode haplotypes, these separated by 1.3%. Specimens with both haplotypes are found throughout its range and display no consistent differences in habitus or male or female genitalia. Similarly, the distinctive red morph from San Benito County flies with typical black specimens with which they are indistinguishable by barcodes or genitalia.

##### Distribution and biology.

*Aseptis fanatica* is known from Washington, Oregon, California, and Baja California Norte, Mexico. It flies in many different habitats like brush land and open forest in southern California mostly at 1000–2000 m but occurs at lower elevations farther north. The flight period is from early May to August in the south and in mid-summer in the Cascades. It can be abundant. In the Pacific Northwest, *Aseptis fanatica* feeds on species of Ericaceae such as madrone (*Arbutus menziesii* Pursh.) and various species of manzanitas and bearberry (*Arctostaphylos* spp.) (Miller & Hammond 2003, as *Aseptis ethnica*). Bearberry (*Arctostaphylos uva-ursi* (L.) Spreng.) is probably the only suitable foodplant for this species in the Washington Cascades.

##### Discussion.

Prior to its description in 2006, this species was thought to represent the southern California form of *Aseptis ethnica*. In fact, the holotype of *Hadena ethnica* is quite similar to *Aseptis fanatica* from San Diego County.

The mixed red and black population from San Benito County is a unique phenomenon. The red color might be due to a gene mutation, but could also be adaptive. Many noctuids that feed on *Arctostaphylos* as larvae are a similar red color, including *Mesogona rubra* Hammond & Crabo in the subtribe Xylenina.

The name *fanatica* means fanatic and was selected as the antithesis of *ethnica* (heathen). At the time, fanatic had a less sinister meaning than it does in today’s world.

### *Aseptis lichena* species group

The *Aseptis lichena* species group consists of two closely related species from the mountains of central and southern California. They are unique in the genus in that the forewing is mottled olive, darker olive green in *Aseptis lichena* (Barnes & McDunnough) and paler yellow tan in *Aseptis pseudolichena* Mustelin & Leuschner. The male valves have a unique acute spine from the ventral cucullus and lack a digitus. The vesicas have a single long apical cornutus. The female bursae are indistinguishable, with large corpora bursae without distinct signa and rounded appendices bursae. The CO1 barcode sequences of the two species differ by approximately 2.3%.

#### Aseptis lichena

Taxon classificationAnimaliaLepidopteraNoctuidae

(Barnes & McDunnough, 1912)

**Figures 19–30.**
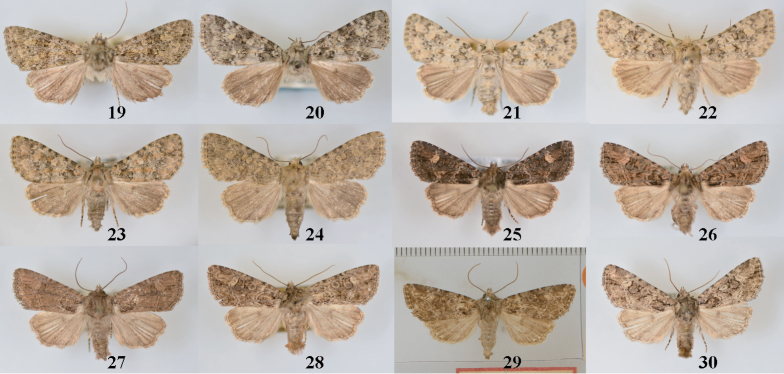
*Aseptis* adults. **19** *Aseptis lichena*, male (Plumas Co., CA) **20** *Aseptis lichena*, female (Tuolumne Co., CA) **21** *Aseptis pseudolichena*, male (Los Angles Co., CA) **22** *Aseptis pseudolichena*, female Paratype (San Diego Co., CA) **23** *Aseptis pseudolichena*, male (San Diego Co., CA) **24** *Aseptis pseudolichena*, female (Ventura Co., CA) **25** *Aseptis binotata*, male (Kittitas Co., WA) **26** *Aseptis binotata*, male (San Diego Co., CA) **27** *Aseptis binotata*, male (Ventura Co., CA) **28** *Aseptis binotata*, male (Okanogan Co., WA) **29** *Aseptis binotata*, female Type of *genitrix* (Nevada) **30** *Aseptis binotata*, male (Summit Co., CO).

Figs 19 , 20 , 64 , 81

Andropolia lichena Barnes & McDunnough, 1912b: 17.

##### Type material.

**Holotype** female [USNM, examined]. Type locality: Deer Park Springs, Lake Tahoe, California.

##### Diagnosis.

A medium-sized noctuid with a wingspan of 35.5±1.8 mm (n=8; range 33–39 mm) with a powdery dark olive-green forewing produced by a mixture of black, green, and yellow scales. *Aseptis lichena* is darker green than *Aseptis pseudolichena*. The male valve of *Aseptis lichena* is nearly straight whereas that of *Aseptis pseudolichena* is bent slightly ventrad at its midpoint. In practice, most specimens can be assigned to a species based on geography, except in an area of overlap at the south end of the Sierra Nevada in Kern and Tuolumne counties. *Aseptis lichena* occurs to the north of this zone. Females are best associated with the males.

##### Distribution and biology.

The relatively few specimens of *Aseptis lichena* we have examined are from south-central California (Tehachapi Mountain Peak, Kern Co.) and north-central California (near Blairsden, Plumas Co., Lake Tahoe, and Yosemite Park). It is also reported from Mount Shasta, Mount Lassen, and other locations in northern California. It flies during mid-summer. The early stages are unknown.

##### Discussion.

This species was described in the genus *Andropolia* Grote, possibly because the holotype is a dark female with a relatively inconspicuous indentation of the hindwing. Specimen labels found by the senior author suggest that McDunnough suspected that this was incorrect. A female collected in Yosemite National Park, Camp 19, on 15 July 1937 by F.L. Cramer has a second label: “McD needs,” a third label: “*Andropolia lichena* B & McD., Det. Dr. J. McDunnough,” and a fourth label: “Probably misplaced in “*Andropolia*” – McD.” Nonetheless, it remained in *Andropolia* until it was associated with *Aseptis* by Mustelin et al. (2000).

#### Aseptis pseudolichena

Taxon classificationAnimaliaLepidopteraNoctuidae

Mustelin & Leuschner, 2000

Figs 21–24 , 56 , 82

Aseptis pseudolichena Mustelin & Leuschner, 2000. In Mustelin et al. 2000: 10.

##### Type material.

**Holotype** male [LACM, examined]. Type locality: East Fork of Woodwardia Camp, San Gabriel Mountains, Los Angeles County, California.

##### Diagnosis.

*Aseptis pseudolichena* is similar in size or slightly smaller than *Aseptis lichena*, wingspan 33.8±1.4 mm (n=25; range 30.5–36.0 mm) and resembles it closely. *Aseptis pseudolichena* is more yellowish as a result of a mixture of pale tan or yellowish scales and scattered tan, olive, and black scales. Some specimens are very pale yellow, others pale tan, and some olive tan. The antemedial and postmedial lines are serrate, and the postmedial line is followed by white and black dots on the veins. A pale subterminal line is usually visible. The orbicular spot is round and filled with ground color, whereas the reniform spot is large, outlined in black and filled with dark scales. A faint pale postreniform patch is present. Males have a pale streak between hindwing veins M1 and M3.

Most *Aseptis pseudolichena* can be separated readily from *Aseptis lichena* by their pale-yellowish to olive-tan color, which is darker olive tan in *Aseptis lichena*; however, dark specimens of *Aseptis pseudolichena* may not be distinguishable without dissection. The male genitalia are similar to those of *Aseptis lichena*, but differ in that the valves are angled ventrad at mid-length and the cucullus is smaller. Most specimens can be assigned to a species based on locality as described under *Aseptis lichena*.

##### Distribution and biology.

*Aseptis pseudolichena* is endemic to southern California with records from San Diego, Riverside, Los Angeles, Ventura, San Bernardino, and Tuolumne counties. It may overlap with *Aseptis lichena* in Kern and Tuolumne counties. *Aseptis pseudolichena* is found in open pine and oak forest, open areas with grass and scrub, and foothill chaparral. It seems to be most common on the desert side of the mountain peaks, and can be locally abundant. It flies from June to August depending on elevation. A number of specimens in the Los Angeles County Museum were raised from larva on *Ribes malvaceum* Sm. (Grossulariaceae). The pupa was described and figured by Comstock (1955) under the name *Andropolia lichena*.

#### Aseptis binotata

Taxon classificationAnimaliaLepidopteraNoctuidae

(Walker, 1865)

Figs 25–30 , 31–34 , 66 , 83

Mamestra binotata Walker, 1865a: 663.
Miana rubiginosa Walker, 1865a: 675.
Hadena extersa Walker, 1865b: 728.
Taeniocampa paviae Strecker, 1874: 94, **syn. n.**
Hadena curvata Grote, 1874b: 157, **syn. n.**
Hadena genitrix Grote, 1878: 237, **syn. n.**
Hadena inconspicua Smith, 1893: 142, **nomen nudum**
Hadena dilara Strecker, 1898: 7, **syn. n.**
Hadena bultata Smith, 1906: 228, **syn. n.**
Trachea cara Barnes & McDunnough, 1912c: 52, **syn. n.**

##### Type material.

*Mamestra binotata*: **Holotype** male [BMNH, photograph examined]. Type locality: Vancouver Island, British Columbia. *Miana rubiginosa*: **Holotype** male [BMNH, not examined]. Type locality: Vancouver Island, British Columbia. *Hadena extersa*: **Holotype** male [BMNH, photograph examined]. Type locality: Vancouver Island, British Columbia. *Taeniocampa paviae*: **Syntypes** [Strecker coll., not examined]. Type locality: California. *Hadena curvata*: **Holotype** female [BMNH, photograph examined]. Type locality: Mendocino, California. *Hadena genitrix*: **Holotype** female [BMNH, photograph examined]. Type locality: Nevada. *Hadena inconspicua*: **Lectotype** male designated by Todd (1982) [USNM, examined]. Type locality: California. *Hadena dilara*: **Holotype** female [FMNH, photograph examined]. Type locality: Colorado. *Hadena bultata*: **Lectotype** male designated by Todd (1982) [AMNH, examined]. Type locality: Glenwood Springs, Colorado. *Trachea cara*: **Syntypes** [USNM, examined]. Type locality: Eureka and Provo, Utah.

##### Diagnosis.

*Aseptis binotata* is a common medium-sized member of the genus with a wingspan of 32.5±1.3 mm (n=25; range 29.5–35.0 mm). It is the most variable *Aseptis* with respect to forewing color and pattern strength. It may be brownish, warm dark brown, pale to medium gray brown, yellowish light brown, or reddish brown depending on locality. The most noticeable marking in dark specimens is the large pale yellowish postreniform patch, which is bisected by the dark postmedial line. The antemedial line is strongly convex laterally and is filled with light tan. Black forewing markings include a series of wedges near the outer margin below the apex, the outlines of the three forewing spots, and in most specimens a short black basal dash. Pale specimens can be washed out or have contrasting dark markings.

**Figures 31–42.**
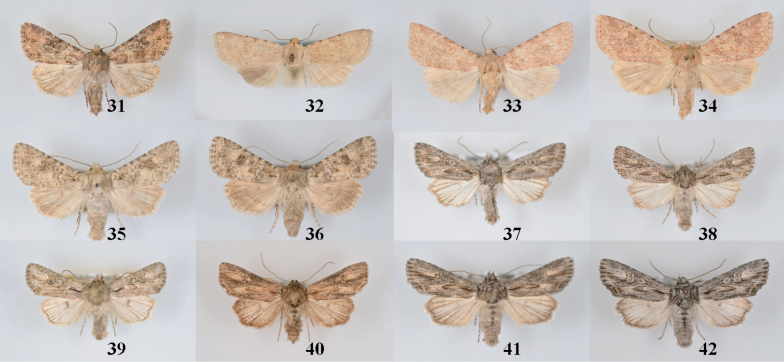
*Aseptis* adults. **31** *Aseptis binotata*, male (Laramie Co., WY) **32** *Aseptis binotata*, female (Glenwood Springs, CO) **33** *Aseptis binotata*, male (San Juan Co., UT) **34** *Aseptis binotata*, female (Garfield Co., UT) **35** *Aseptis catalina*, male (San Diego Co., CA) **36** *Aseptis catalina*, female (San Diego Co., CA) **37** *Aseptis serrula*, male (Imperial Co., CA) **38** *Aseptis serrula*, female (San Bernardino Co., CA) **39** *Aseptis torreyana*, female (San Diego Co., CA) **40** *Aseptis susquesa*, male (San Diego Co., CA) **41** *Aseptis susquesa*, male (San Diego Co., CA) **42** *Aseptis susquesa*, female (San Diego Co., CA).

There is striking variation in this species, both within populations and over larger distances. Specimens from the coastal region of the Pacific Northwest are fairly uniform with a warm dark brown forewing with distinct maculation (Fig. 25). Southern California specimens of *Aseptis binotata* range from nearly as dark (Fig. 26) as Northwestern ones to pale reddish, tan, or pale gray brown with a less contrasting postreniform patch (e.g., Fig. 27). These pale forms were described as *Hadena curvata* Grote and *Taeniocampa paviae* Strecker. Populations from drier habitats east of the coastal mountains also tend to be pale and more uniform in color, often with gray tones (Figs 28, 29). Populations from near the Rocky Mountains are also variable, mostly dull gray brown (Figs 30, 31, but those from areas of Colorado, Utah, and New Mexico with reddish substrate are yellow tan to orange tan, often with reduced dark patterns (Figs 32–34). These colorful morphs were described as *Hadena dilara* Strecker and *Hadena bultata* Smith.

The male genitalia of *Aseptis binotata* have a valve with a curved upright ampulla, a long downwardly curving digitus, and a weak constriction at the base of the cucullus. The vesica is average in size for the genus with a single long apical cornutus and two small diverticula located at the base and mid-portion. The female bursa is 1.3× as long as wide, has distinct signa, and a short slightly bent appendix bursae with a crenulate shape.

Most specimens of *Aseptis binotata* can be recognized, despite the variation in this species, by their brownish color and pale postreniform patch. They are most likely to be confused with *Paraseptis adnixa*, which occurs with it in the Pacific Coast states. The basal dash of *Aseptis binotata* is short, not reaching the antemedial line, whereas that of *Paraseptis adnixa* extends to the antemedial line. Structurally, the male vesica of *Paraseptis adnixa* is coiled and lacks a large apical cornutus, and the female ductus bursae has a sclerotized plate in its posterior wall, unlike those of *Aseptis*.

##### Distribution and biology.

This species is widespread in western North America west of south-central Alberta, Wyoming, and Nebraska. Along the Pacific Coast it occurs from northern Mexico to south-central British Columbia. It can be found from sea level to altitudes over 2000 m in a variety of habitats from dense forest to shrub desert. In the south the flight begins in March at low elevations and in April to July in the mountains. In the north the flight begins later and lasts into August. The larva is green with a white and red lateral stripe. It feeds on numerous woody shrubs, including *Ribes* spp. (Grossulariaceae), *Oemleria cerasiformis* (Torr. & Gray ex Hook. & Arn.) (Rosaceae), *Salix* spp. (Salicaceae), *Acer* spp. (Aceraceae), and *Symphoricarpos* spp. (Caprifoliaceae) (Miller and Hammond 2000, Miller and Hammond 2003). Hampson (1908) mentions *Adenostoma fasciculatum* Hook. & Arn. (Rosaceae) as a foodplant and Crabtree and Leuschner (2000) found larvae on *Prunus emarginata* (Douglas ex Hook.) D. Dietr., Prunus virginiana (L.) var. demissa (Nutt.) Torr., and *Prunus subcordata* Benth. (Rosaceae).

##### Discussion.

Despite the superficial variability of *Aseptis binotata* the male and female genitalia and CO1 barcodes of this species are remarkably uniform. Barcodes from parts of its northern distribution differ by circa 1% from other populations but there is almost no variation elsewhere, including the reddish forms in the Southwest or the variable California populations. We do not advocate the use of subspecies in *Aseptis binotata* because the variation is clinal and because of significant variation in color within populations.

The species name *genitrix* has often been misspelled as “*genetrix*”.

#### Aseptis catalina

Taxon classificationAnimaliaLepidopteraNoctuidae

(Smith, 1899)

Figs 35 , 36 , 67 , 84

Hadena catalina Smith, 1899: 261.

##### Type material.

*Hadena catalina*: **Lectotype** [USNM, examined]. Type locality: Catalina Springs, Arizona.

##### Diagnosis.

An average or slightly smaller than average *Aseptis* species, wingspan 31.8±0.9 mm (n=25; range 30–33 mm), with a powdery pale yellow-tan forewing with patchy contrasting darker gray markings including the filling of the reniform spot and the adjacent medial area. The postreniform patch is large but only slightly lighter in color than the fold portion of the medial area and the filling of the lines. The basal and postmedial areas are darker. The reniform spot is large and it and the claviform spot are filled with dark gray. The antemedial and postmedial lines are black, filled with pale cream and the postmedial line is often followed by black and white dots on the veins. The subterminal area is pale cream, the terminal line is a series of black spots and the fringe is checkered. There is some variation in the color and tone of the ground color, which can be very pale cream or more tan colored. Although the pattern is complete, the maculation is usually indistinct.

The male genitalia are similar to those of *Aseptis binotata* but the valve lacks the digitus, the ampulla of the clasper is thicker and is oriented parallel to the dorsal valve margin, and the dorsal apex of the cucullus is pointed slightly. The aedeagus and vesica are like those of *Aseptis binotata* except for the presence of a granulose area on the ventral apex of the aedeagus. In the female, the corpus bursae is oblong, circa 2.4× as long as wide, with a curved appendix bursae of nearly the same size. The papillae anales are unique in the genus in that they are covered by sparse short needle-like setae with brush-like very dense basal setae condensed into patches on the ventral sides.

*Aseptis catalina* can be recognized by its patchy pale-tan and gray forewing and is unlikely to be confused with other *Aseptis*; however, they resemble superficially *Tridepia nova* (Smith) and *Scotogramma densa* Smith, both in the Hadenini, and are often mixed with them in collections. It is easily separated from them by the eyes, naked in *Aseptis*, but covered in fine hairs in the two hadenines, as well as by the lack of a notched hindwing in these species.

##### Distribution and biology.

This species occurs in deserts of Arizona, California and Baja California, Mexico. Most specimens are from the western edge of the Colorado Desert in San Diego, Imperial, and Riverside counties, California, but there are colonies throughout the Colorado, Mojave, and Sonora deserts. Like many desert insects, the flight period depends on winter rainfall and is early, generally early March to April. The food plants and immature stages are unknown.

#### Aseptis serrula

Taxon classificationAnimaliaLepidopteraNoctuidae

(Barnes & McDunnough, 1918)

Figs 37 , 38 , 68 , 85

Trachea serrula Barnes & McDunnough, 1918: 104.

##### Type material.

**Holotype** male [USNM, examined]. Type locality: Palm Springs, Riverside County, California.

##### Diagnosis.

This below-average-sized *Aseptis*, wingspan 31.7±1.2 mm (n=19; range 29–34), is the only one with a serrate male antenna. The forewing is relatively narrow, powdery gray, with the pointed black claviform spot as the most prominent mark. The dark reniform and orbicular spots are less prominent, the basal, antemedial, and postmedial lines are faint or absent, and the subterminal line is often evident as a pale W-mark on veins M3 and CuA1. The postreniform patch is relatively small, and the medial area is often lighter than the ground color near the claviform spot. The hindwing is off-white with dark veins and terminal area in males and darker gray with light base and dark veins in females.

The male uncus is unique in that the subbasal segment is expanded and dorsoventrally flattened to an elongate rhomboid shape with a slight constriction at the end of the swollen segment. The valve is most similar to those of *Aseptis catalina* and *Aseptis torreyana*, with a gently-curving S-shape, a curved ampulla of the clasper that is directed distally, a narrow pointed digitus, and a slightly pointed cucullus. The vesica is like that of *Aseptis binotata*. In the female, the papillae anales are unique in being covered dorsally by short scales with a rugose medial margin. The bursa copulatrix is nearly round with strong signa and the appendix bursae is as long as the corpus bursae and curved leftward and dorsad from its origin.

Males of *Aseptis serrula* are distinguished easily by the serrate antenna, filiform in other *Aseptis*. Females are readily identified by their unique papillae anales. Many gray desert noctuids resemble *Aseptis serrula*, including several species of Hadenini with which it is often confused. Differences between *Aseptis* and hadenines are described under *Aseptis catalina*.

##### Distribution and biology.

This is a species of the lower mountain-desert transition zone and high desert and has been collected in the Mojave, Colorado, and Sonora deserts of southeastern California, Nevada, Arizona, and Baja California. It flies during the desert spring, between March and early May depending on winter rainfall. Its host plants and immature stages are unknown.

#### Aseptis torreyana

Taxon classificationAnimaliaLepidopteraNoctuidae

Mustelin, 2006

Figs 39 , 69

Aseptis torreyana Mustelin, 2006: 29.

##### Type material.

**Holotype** male [SDNHM, examined]. Type locality: Torrey Pines State Reserve, La Jolla, California.

##### Diagnosis.

This is the smallest and rarest *Aseptis* (wingspan 27.5 mm). It has a pale buff forewing overlaid with pale gray scales and marked with black basal dash and outlines of the three spots. The reniform spot is the largest and is filled with dark scales. The pale postmedial line is barely visible while the antemedial line is missing. The strong black basal dash gives the impression of a small pale *Paraseptis adnixa*.

Males of *Aseptis torreyana* have a narrow S-shaped valve with an ampulla of the clasper that is oriented toward the apex of the valve, a small digitus that arises near the ventral attachment of the clasper and is oriented perpendicular to the valve, and a small rounded cucullus. The aedeagus and vesica resemble those of *Aseptis binotata*. The female is unknown.

This species is unlikely to be confused with any other *Aseptis* because of its small size and isolated habitat. It resembles a pale *Aseptis serrula*, although the markings of *Aseptis torreyana* are more distinct. The male antenna of *Aseptis torreyana* is filiform rather than serrate. *Aseptis torreyana* is also reminiscent of *Aseptis characta* (Grote) but is easily distinguished from it by the present of a digitus on the male valve.

##### Distribution and biology.

This species is known only from the south side of the sea level salt marsh estuary of the Torrey Pines State Reserve. The habitat is most likely salt marsh, although it could be coastal chaparral. The capture date of April 21 is in line with the spring flight period of most *Aseptis* species in southern California. The foodplant is unknown.

##### Discussion.

*Aseptis torreyana* is enigmatic because of its rarity. It was discovered in a small isolated coastal chaparral remnant bordering the salt marsh within Torrey Pines State Reserve, San Diego, California. Only two males were ever found, both at this locality on the same night, despite a two-year moth survey by Norris Bloomfield and the senior author. The latter also ran a blacklight nearly every night for five years at the rim of the Peñasquitos Canyon, which runs into the same estuary 5 miles east of the type locality without finding additional specimens. Similarly, none were found at the nearby Miramar Air Station that was surveyed extensively for Lepidoptera for years with same traps (Brown and Bash 2000). Taken together, it appears that *Aseptis torreyana* is very local, perhaps tied to a food plant in the salt marsh. Another example of such a restricted species is *Orthomoia bloomfieldi* Mustelin that was described from 30 specimens from a single locality during the Miramar study.

#### Aseptis susquesa

Taxon classificationAnimaliaLepidopteraNoctuidae

(Smith, 1908)

Figs 40–42 , 70 , 86

Hadena susquesa Smith, 1908: 116.
Trachea monica Barnes & McDunnough, 1918: 104, **syn. n.**

##### Type material.

*Hadena susquesa*: **Lectotype** male designated by Todd (1982) [AMNH, examined]. Type locality: Claremont, California. *Trachea monica*: **Holotype** male [USNM, examined]

##### Diagnosis.

This is a slender-winged small to medium-sized *Aseptis*, wingspan 31.4±1.2 mm (n=25). It is readily recognized by its streaky medium-gray to dark-brown gray forewing with streaks of warm light orange tan to yellow tan at the postreniform patch, in the fold, and in the large pointed claviform spot. A thin tan line parallels the margin near the anal angle. The reniform and orbicular spots are outlined in black with paler peripheral and darker central scaling. The distal forewing is streaky due to black veins and pale-gray scales abutting R5, M1, M3, and CuA1. The transverse lines are obsolete. The hindwing is light whitish gray with brown-gray marginal shading and dark veins, darker in females. *Aseptis susquesa* from coastal California tend to be rustier than those from inland locations.

The male valve of *Aseptis susquesa* is similar to that of *Aseptis binotata*, although the cucullus is larger. The uncus is thin, the valve has an upright ampulla, the oblique digitus is long and pointed, and the cucullus is approximately 1.5× as wide as the valve and slightly pointed. The vesica has two small diverticula and a single apical cornutus. The female genitalia has a papilla analis covered by similar-length needle-like setae and sparse hair-like basal setae; the corpus bursae is relatively small and short, 1.25× as long as wide, with a similar sized appendix bursae that is laterally compressed and asymmetrically bulging ventrally.

No other *Aseptis* is streaked gray with patches of light orange or rusty color. *Aseptis susquesa* is most similar to *Aseptis serrula* and flies with it. It is similar gray but has light color restricted to a small postreniform patch. Males of these species are easily distinguished by their antennae

##### Distribution and biology.

*Aseptis susquesa* is known from Arizona, California, and Baja California, Mexico, at least as far south as Ensenada. Most records of the rusty coastal form are from San Diego, Riverside, and Los Angeles counties, California, where it inhabits coastal chaparral and canyons from late March to early June. The grayer inland form is found in the Mojave and Colorado deserts of southern California and in the Sonora Desert of Arizona. *Aseptis susquesa* prefers rocky areas in the mountain-desert transition zone and high desert. The larva is dark green marked with white and feeds on *Artemisia californica* Less. (Asteraceae) (unpublished) and *Ericameria laricifolia* (A. Gray) Shinners (Asteraceae) (Crumb 1956).

##### Discussion.

In their original description of *Trachea monica* from Redington, Arizona, Barnes and McDunnough (1918) pointed out that it is similar to *Hadena susquesa*, described previously from Claremont, California, and might be a gray inland form of it. In support of that notion, the lectotype of *Hadena susquesa* is rather gray whereas some desert specimens from farther inland have considerable rusty brown demonstrating variability and overlap. The male genitalia of these forms are virtually uniform throughout the range. The CO1 barcodes, including specimens typical of coastal and inland forms, vary by less than 0.8%. For these reasons, we treat *Trachea monica* Barnes & McDunnough as a junior subjective synonym of *Hadena susquesa* Smith.

#### Aseptis fumosa

Taxon classificationAnimaliaLepidopteraNoctuidae

(Grote, 1879)

**Figures 43–58.**
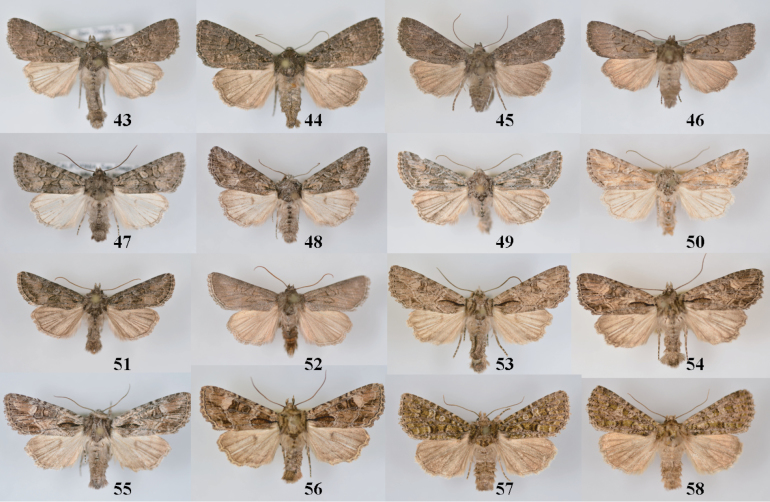
*Aseptis* adults. **43** *Aseptis fumosa*, male (San Diego Co., CA) **44** *Aseptis fumosa*, female (San Diego Co., CA) **45** *Aseptis perfumosa*, female (San Diego Co., CA) **46** *Aseptis perfumosa*, male (San Diego Co., CA) **47** *Aseptis characta*, male (San Diego Co., CA) **48** *Aseptis characta*, male (Summit Co., CO) **49** *Aseptis characta*, male (Yakima Co., WA) **50** *Aseptis characta*, male (Lost River, AB) **51** *Aseptis characta*, male (Seton Lake, BC) **52** *Aseptis characta*, male (Prineville, OR) **53** *Paraseptis adnixa*, male (San Diego Co., CA) **54** *Paraseptis adnixa*, male (San Diego Co., CA) **55** *Paraseptis adnixa*, female (Inyo Co., CA) **56** *Paraseptis adnixa*, male (Langley, BC) **57** *Viridiseptis marina*, male (San Diego Co., CA) **58** *Viridiseptis marina*, male (San Diego Co., CA).

Figs 43 , 44 , 71 , 87

Hadena fumosa Grote, 1879: 205.

##### Type material.

**Holotype female** [BMNH, photograph examined]. Type Locality: Colorado.

##### Diagnosis.

A medium-sized *Aseptis*, wingspan 34.9±1.5 mm (n=25; range 31.5–38 mm) with a very dark forewing and whitish hindwing with black veins in males and darker gray hindwing in females. The forewing is uniform smoky dark blackish brown with brown filling of the antemedial and postmedial lines that is most evident as dots on the costa. The weakly figure-eight shaped reniform spot, orbicular spot, and short claviform spot are black filled with ground color or slightly darker scales. The male hindwing is pearly gray distal to the spot that accentuates the vein asymmetry. The hindwing of the female is smoky dark with dark but less conspicuous veins.

The male genitalia of *Aseptis fumosa* are unique in several respects. The valve extends nearly 90° lateral from its support and is a simple strap with a narrow base, very weak sacculus, convex ventral mid-portion, and undifferentiated cucullus; the clasper is near the base with a short straight ampulla parallel to the costa, and the digitus is absent. The uncus is thin and cylindrical. The penicillus is weak with a pointed dorsal margin. The vesica is similar to that of *Aseptis binotata* but bears an additional thick-based thorn-like mesial cornutus.

The female has a papilla analis covered with short needle-like setae and sparse basal hairs. The corpus bursae is fairly short, 1.5× as long as wide, with strong signa and the appendix bursae is box shaped and laterally compressed.

*Aseptis fumosa* males are distinctive due to the combination of blackish forewing and white hindwing with dark veins. Despite this, *Aseptis fumosa* is often confused with *Aseptis perfumosa* in collections. Females of both species have dark hindwings and are less easily separated. *Aseptis fumosa* is usually larger with a broader forewing and its spots, especially the claviform spot, are less prominent than in *Aseptis perfumosa*. *Aseptis fumosa* females can also be confused with dark species in the *Aseptis fumeola* species group such as *Aseptis ethnica* and *Aseptis fanatica*. The shapes of the bursae are distinctive. The blunt rounded appendix bursae of *Aseptis fumosa* distinguishes it from all of the look-alikes.

**Figures 59–65.**
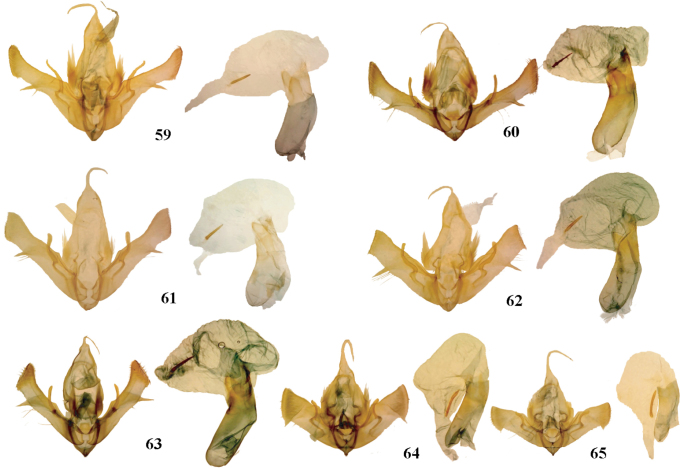
*Aseptis* male genitalia. **59** *Aseptis fumeola* **60** *Aseptis ethnica* **61** *Aseptis murina* **62** *Aseptis ferruginea* **63** *Aseptis fanatica* **64** *Aseptis lichena* **65** *Aseptis pseudolichena*.

##### Distribution and biology.

*Aseptis fumosa* is widespread in western North America and is known from western Canada, Washington, Nebraska, Colorado, Utah, Arizona, Nevada and California. It occurs in a variety of diverse habitats including coast chaparral, dry conifer forest, and shrub steppe; it is not found in mesic forests. The flight begins in April or May and lasts to July. The larva is smooth green with a white subdorsal stripe and broad red and white lateral stripe (Miller and Hammond 2003). It has been reared on *Purshia tridentata* (Pursh) DC., *Cercocarpus* sp. and *Adenostoma fasciculatum* Hook. & Arn. (all Rosaceae) (Crumb 1956, Crabo et al. 2012). At higher altitudes in the Cascades and in southwestern Oregon it feeds on *Ceanothus integerrimus* Hook. & Arn. (Rhamnaceae) (Miller and Hammond 2000).

##### Discussion.

The simple valve of *Aseptis fumosa* is similar to that of *Aseptis characta*. Both of them also have multiple cornuti on the vesica. Although these derived states of the valve suggest a close relationship, their female bursae differ in shape and that of *Aseptis characta* lacks signa.

#### Aseptis perfumosa

Taxon classificationAnimaliaLepidopteraNoctuidae

(Hampson)

Figs 45 , 46 , 72 , 88

Trachea perfumosa Hampson, 1918: 131.

##### Taxonomy.

The type material of *Trachea perfumosa* Hampson, the holotype female and two paratype females, was originally part of the type series of *Trachea fumeola* Hampson 1908. In fact, the female later selected as the *Trachea perfumosa* holotype was depicted on plate 112 as *Trachea fumeola*. The male and females were described in separate paragraphs and the male was selected as the type of *Trachea fumeola*. Hampson later realized that these females and the male type were separate species, naming the females *Trachea perfumosa* Hampson, 1918. The 1918 work lacks a description—initially leading us to suspect that *Trachea perfumosa* is a nomen nudum—but instead references the female *Trachea fumeola* description and illustration in the earlier work. This indication thereby validates the name.

##### Type material.

**Holotype**: female [BMNH, photograph examined]. Type locality: USA, California.

##### Diagnosis.

This is a small dark *Aseptis* with a wingspan of 32.7±1.2 mm (n=25; range 30.5–34.5 mm). The body appears short and stout and the wings short and stubby. The forewing is slightly mottled dark gray brown, almost black in some specimens, often with a few grayish, brownish, olive, or reddish scales in the medial area around the velvety black spots of which the acute claviform spot is usually the most prominent. Less conspicuous forewing markings include a short black basal dash, incomplete faint wavy basal, antemedial and postmedial lines filled with brown, and irregular complete brown subterminal line. The hindwing of both sexes is dark grayish brown with inconspicuous veins.

Males of *Aseptis perfumosa* are separated easily from all other species of *Aseptis* by the short spike-like digitus that arises near the ventral margin and is perpendicular to it. The 90° basal bend of the vesica and basally-constricted medial diverticulum are also diagnostic. Females are identified by the thin flange on the tip of each papilla analis and by the bilobed appendix bursae.

Superficially, *Aseptis perfumosa* is most similar to *Aseptis fumosa*. *Aseptis perfumosa* tends to be smaller and narrower winged than *Aseptis fumosa*. Males are separated easily by hindwing color, dark in *Aseptis perfumosa* and pale in *Aseptis fumosa*. Females can often be separated based the maculation as the claviform spot is usually the most prominent feature on *Aseptis perfumosa*, whereas the postmedial line is clearer in *Aseptis fumosa*. The correct identity can be confirmed by examining the tips of the ovipositors under magnification.

**Figures 66–75.**
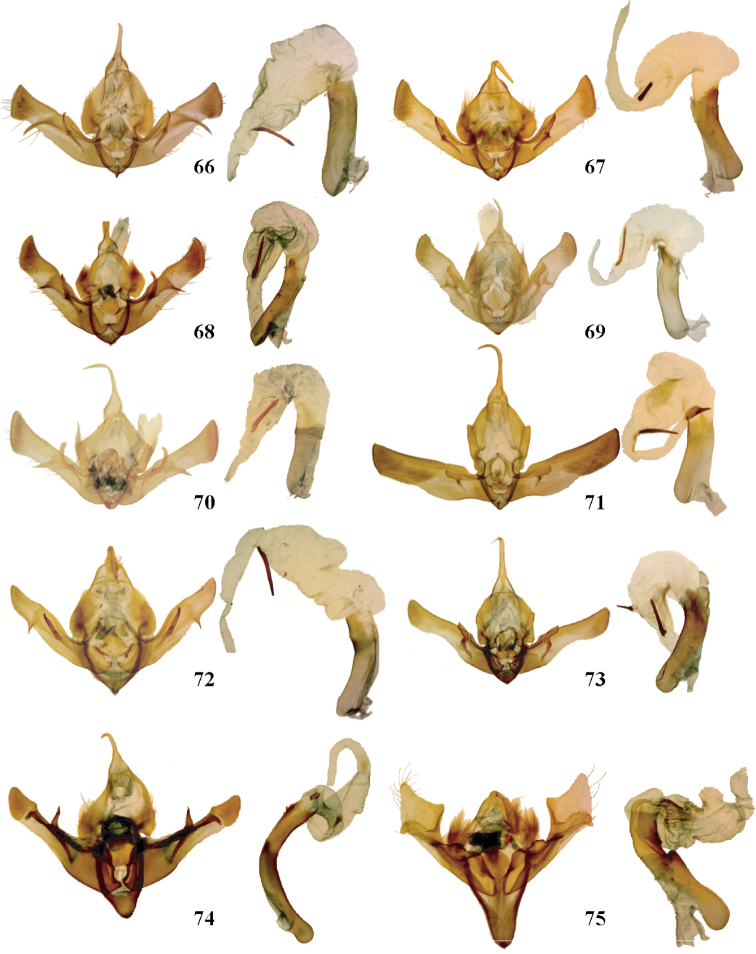
*Aseptis*, *Paraseptis*, and *Viridiseptis* male genitalia. **66** *Aseptis binotata* **67** *Aseptis catalina* **68** *Aseptis serrula* **69** *Aseptis torreyana* **70** *Aseptis susquesa* **71** *Aseptis fumosa* **72** *Aseptis perfumosa* **73** *Aseptis characta* **74** *Paraseptis adnixa* **75** *Viridiseptis marina*.

**Figures 76–91.**
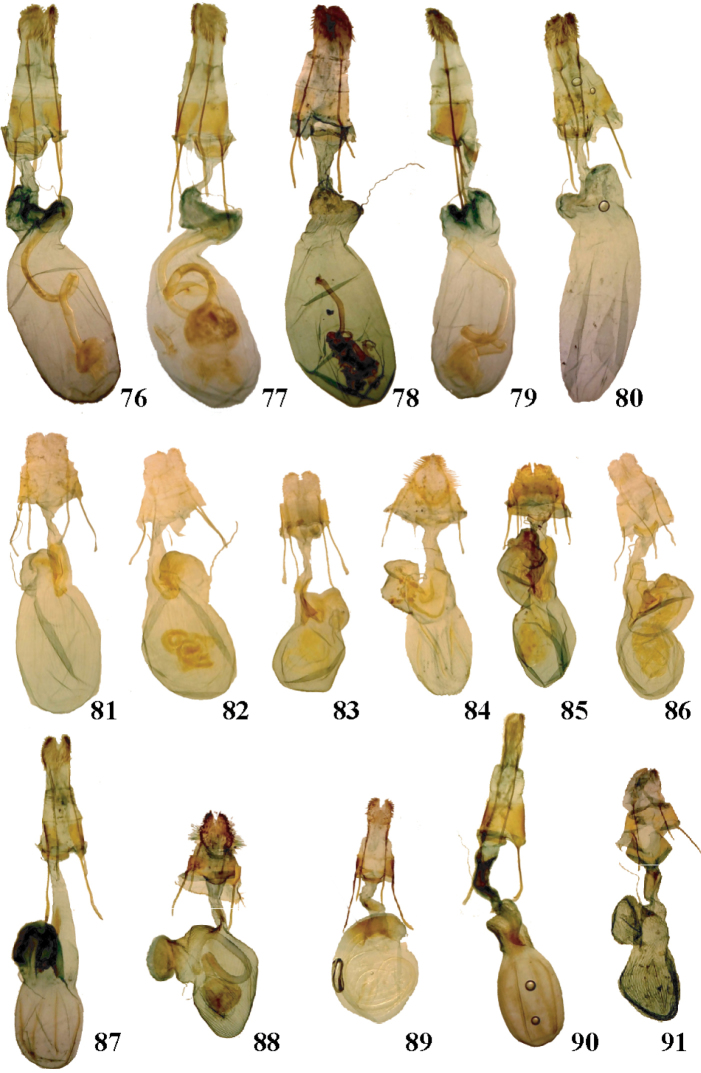
*Aseptis*, *Paraseptis*, and *Viridiseptis* female genitalia. **76** *Aseptis fumeola* **77** *Aseptis ethnica* **78** *Aseptis murina* **79** *Aseptis ferruginea* **80** *Aseptis fanatica* **81** *Aseptis lichena* **82** *Aseptis pseudolichena* **83** *Aseptis binotata* **84** *Aseptis catalina* **85** *Aseptis serrula* **86** *Aseptis susquesa* **87** *Aseptis fumosa* **88** *Aseptis perfumosa* **89** *Aseptis characta* **90** *Paraseptis adnixa* **91** *Viridiseptis marina*.

##### Distribution and biology.

*Aseptis perfumosa* is endemic to southern California where it occurs in many habitats such as coastal chaparral and canyons, urban areas, brush land, and open oak forest from sea level to 2000 m. It is often very common and can be the most abundant noctuid species. The peak of its flight is early April to early June in coastal areas and a little later at higher elevations. The larva and pupa are described and figured by Comstock (1940b). The green larvae were found and reared on Manzanita (*Arctostaphylos* spp.).

##### Discussion.

Hampson (1918) gives the expanse of the type of *Trachea perfumosa* as 36 mm. This is larger than any *Aseptis perfumosa* specimens examined by us. This led us initially to question whether he might have described a female of the larger species *Aseptis fumeola* rather than *Aseptis perfumosa*, given that the latter species was named from a specimen in the type series of the former species. While enquiring about the types at the BMNH, Alberto Zilli (pers. comm. 2015) explained that Hampson’s wingspans are almost always greater than those of the actual moth because of his method of measurement. He measured from the pin to the apex of the forewing and doubled the result. While the actual wingspan of the female type of *Trachea perfumosa* is 33.5 mm, a normal size for the species, the result using Hampson’s method yields the published result of 36 mm.

#### Aseptis characta

Taxon classificationAnimaliaLepidopteraNoctuidae

(Grote, 1880)

Figs 47–52 , 73 , 89

Hadena characta Grote, 1880: 243.
Hadena luteocinerea Smith, 1900: 468.
Hadena erica Smith, 1905: 258.
Hadena pluraloides McDunnough, 1922: 237.

##### Type material.

*Hadena characta*: **Holotype** female [BMNH, photograph examined]. Type locality: Nevada. *Hadena luteocinerea*: **Holotype** male [USNM, examined]. Type Locality: Montana. *Hadena erica*: **Lectotype** male designated by Todd (1982) [AMNH, examined]. Type Locality: Stockton, Utah. *Hadena pluraloides*: **Holotype** female [CNC, examined]. Type Locality: Lethbridge, Alberta.

##### Diagnosis.

This is a narrow-winged smaller *Aseptis*, wingspan 32.0±1.2 mm (n=25; range 29.5–35 mm), with complete forewing pattern of typical lines and spots. The forewing is ash gray to pale tan, often darker gray in the medial area and with variable olive-gray, tan, or orange-tan patches. The basal, antemedial and postmedial lines are double, dark gray filled with pale gray. The postmedial area is lighter with a shade preceding the pale subterminal line and a number of black wedges between the veins, The three spots are outlined in black and are filled with the ground color and, except the claviform spot, peripheral lighter gray scales. The hindwing is gray, pale gray, or white with dark discal spot, veins, and terminal line, darker in females.

*Aseptis characta* is geographically variable, appearing slightly different in each region. In extreme southern California it is relatively smooth gray with a white hindwing (Fig. 47), becoming more powdery with a gray hindwing in Los Angeles and San Bernardino counties and more mottled in the Sierra Nevada. In the Pacific Northwest it is usually darker gray with variable subtle olive or brown shades on the forewing and a fuscous hindwing (Figs 49 and 51), although populations from the Blue Mountains of Washington and Oregon resemble those from the Sierra Nevada. On the Great Plains *Aseptis characta* is lighter, often pale tan with a warm orange cast (Fig. 50).

The male valve is strap-like with a slight S-shape, with a very weak sacculus, slightly expanded cucullus with a rounded apex, rod-like straight ampulla oriented parallel to the costa, and no digitus. The vesica is similar to that of *Aseptis binotata* but has one or two additional spine-like cornuti on its mid-portion. The female has a papilla analis covered densely with short needle-like setae and sparse basal hairs, a rounded corpus bursae lacking signa, and a short appendix bursae that barely changes the outline of the bursa.

*Aseptis characta* can be identified by its small size, mottled gray forewing, and complete pattern of lines and spots. The male is the only *Aseptis* with two or three slender cornuti on the vesica and the female is the only one with an immaculate corpus bursae and weak appendix bursae. This species is similar to several species of *Lacinipolia* McDunnough in the Eriopygini, especially *Lacinipolia pensilis* (Grote), and is often intermixed with them in collections. They can be distinguished by the hindwing notch of *Aseptis* and minute hairs on the eyes of *Lacinopolia*.

##### Distribution and biology.

*Aseptis characta* is widespread in western North America in the western Great Plains, Great Basin, and Pacific regions from British Columbia, Alberta, and Saskatchewan to Colorado, Utah, northern Arizona and southern California. It does not occur on the immediate Pacific Coast north of central California. It flies in dry habitats like sagebrush steppe, juniper woodlands, and open forest from sea level to 2500 m and is often common. In southern California it is most often found on the dry side of the mountain ranges, in the mountain-desert transition zone, and in the deserts. Emergence is earliest in xeric habitats, usually April in California and mid-May in the Pacific Northwest. The flight lasts until July to August depending on locality. The striped gray-green and white larva has been found feeding on *Artemisia* spp. (Asteraceae) (Comstock 1955, Crumb 1956).

##### Discussion.

Similarities between the male genitalia of this species and *Aseptis fumosa* are noted under the latter species. The female corpus bursae lacking signa and shallow appendix bursae of *Aseptis characta* are unique.

Given the geographic variability of *Aseptis characta* it is almost surprising that not more names have been given to the various forms. *Hadena erica* Smith was based on specimens from Utah which are bluish ash gray with patches of paler gray and an ochreous tinge on the basal and distal wing similar to Fig. 48. The light orange-tan Great Plains populations were described twice, as *Hadena luteocinerea* Smith from Montana and *Hadena pluraloides* McDunnough from Alberta; both are similar to Fig. 50. The latter name denotes the resemblance to *Euxoa pluralis* (Grote). *Aseptis characta* has a nearly continuous distribution within its range and the different forms are not well enough separated to warrant the use of subspecies.

In contrast to the variation in habitus of this species, the genitalia are uniform. Similarly, the variation of CO1 barcode sequences is small despite a large number of samples (n=67) from throughout its distribution. Multiple slightly different haplotypes cluster within a total range of less than 1%.

#### Genus Paraseptis gen. n.

Taxon classificationAnimaliaLepidopteraNoctuidae

http://zoobank.org/E3418CA4-70AB-4F26-8EE6-ED770970B171

##### Type species.

*Hadena adnixa* Grote.

##### Etymology.

The name *Paraseptis* is derived from *para* meaning next to and *septis* by analogy to *Aseptis*. The name is feminine.

##### Diagnosis.

*Paraseptis* is a monotypic genus whose sole member occurs near the Pacific Coast of North America. It is mottled brown with typical noctuid wing markings, including a basal dash and dark wedges near the outer margin, and a pale off-white to ochre postreniform patch. The outer edge of the hindwing is concave focally between M1 and M3 as in *Aseptis* and *Viridiseptis*. The male genitalia resemble those of *Aseptis* but differ as follows: the valve has a much larger sacculus that extends above the costal margin, a twisted upright ampulla, a rod-like digitus arising near the base of the cucullus from a longitudinal bar near the costa; a long curved aedeagus with distal spine patches of small spines; and a coiled vesica with basal and medial cornuti, but no long apical cornutus (occasional specimens with a minute apical cornutus). The female genitalia are also similar to those of *Aseptis* but differ in having more narrow papillae anales and a strongly sclerotized posterior ductus bursae. CO1 DNA barcodes of *Paraseptis* are not similar to those of *Aseptis* and cluster variably with other genera in the Xylenini when representatives from a large number of species are included in the sample set.

##### Description.

**Adults**: **Head**: Eye rounded, normal sized. Antenna filiform in both sexes. Labial palpus unmodified with longer second segment and short third segment. Frons slightly convex, smooth. *Thorax*: Paired moderate-sized dorsal mesothoracic and smaller metathoracic tufts. Legs without tibial spines; tarsal segments with three rows of short spine-like setae. **Abdomen**: Male with coremata at base of abdomen, complete with lever, pocket, and Stobbe’s gland; proximal segments with weak dorsal tufts. **Forewing**: Venation as typical for subfamily, approximately 0.6× as wide as long, with brownish, black, and off-white scales, appearing mottled brown with black typical noctuid markings including a basal dash. **Hindwing**: Venation trifine as typical for subfamily, M2 weak but usually visible, clustered close to M3 and CuA1; outer margin contour concave between veins M1 and M3. **Male genitalia** (Fig. 74): Tegumen narrow near base of uncus; penicillus large, quadrate. Uncus smoothly downcurved, narrow, tapering smoothly from base to acute tip. Juxta rectangular, circa 2/3× as wide as long. Valve weakly S-shaped, tapered from base to mid-portion then even in width to base of cucullus; sacculus strong, moderately sclerotized, 0.4× valve length and 2× valve width at base of ampulla, extending above costa; cucullus weakly constricted at base and expanded to 1.5–1.7× valve width, slightly rounded with blunt apex, corona of circa 30 claw-like setae; clasper on mesial third, ampulla oriented perpendicular to valve and extending above costa, rod-like with slightly twist to mediolaterally flattened tip; digitus at distal end of an evenly-thick sclerotized ridge located slightly below costa from clasper to digitus origin near cucullus base, rod-like with blunt tip, oriented 45° to valve, ending near ventral cucullus. Aedeagus narrower and more robustly sclerotized than in *Aseptis*, 7× as long as wide, distal half bent ventrad approximately 60°, small patches of small spines near dorsal and ventral apex; vesica slightly wider than aedeagus, coiled 360° to right and ventrad to end posterior, ventrad, and left of aedeagus tip, with subbasal patch of short cornuti on right, single spike-like diverticulum perpendicular to axis on anterior distal third, minute spike-like cornutus directed basad at apex in a few specimens, and very small dome-like mesial diverticulum. **Female genitalia** (Fig. 90): Papillae anales weakly sclerotized, asymmetrically cone-shaped with point near dorsum, circa 1.7× as long as wide, covered posteriorly and apically with short thin setae that are slightly shorter near tip, lacking hair-like basal setae; apophyses moderately long, posterior apophysis 1.7× anterior apophysis; ostium bursae membranous except for thin weak band in ventral wall; ductus bursae tubular, 1× corpus bursae length, proximal 2/3 sclerotized with longitudinal ridge in dorsum, distal 1/3 membranous; corpus bursae ovoid, 0.75× as wide as long, with four long signa evenly spaced on anterior, posterior, and lateral sides; appendix bursae arising from right paramedial ventral posterior corpus bursae, moderately sclerotized, rugose, 0.75× corpus bursae length, conical with 45–60° rightward bend to end ventral to, or slightly to right and ventral to, distal ductus bursae, with ductus seminalis near apex.

##### Discussion.

The structural differences of *Paraseptis* and *Aseptis* are surprising given the nearly identical habitus of *Paraseptis adnixa* and *Aseptis binotata*, which are often mixed in collections.

Several similar features of *Paraseptis* and *Aseptis*, including the hindwing shape and superficial resemblance, suggest that these genera are related closely. The hindwing shape is rare in other genera in the Xylenini. It is a prominent feature of the Eurasian monotypic genus *Atypha* Hübner. The male genitalia of *Atypha pulmonaris* (Esper), illustrated by Fibiger and Hacker (2007), are similar to those of *Aseptis fumosa* and *Aseptis characta* in having a simple valve, horizontal ampulla, and no digitus, suggesting that these three genera might share a common ancestor.

#### Paraseptis adnixa

Taxon classificationAnimaliaLepidopteraNoctuidae

(Grote, 1880) comb. n.

Figs 53–56 , 74 , 90

Hadena adnixa Grote, 1880: 243.
Hadena pausis Smith, 1899: 262, **syn. n.**

##### Type material.

*Hadena adnixa*: **holotype** male [BMNH, photograph examined]. Type locality: Nevada. *Hadena pausis*: **lectotype** male designated by Todd (1982) [USNM, examined]. Type locality: Los Angeles County, California.

##### Diagnosis.

A medium-sized noctuid, wingspan 34.9±1.3 mm (n=25; range 32–37.5 mm), that resembles strongly a narrow-winged *Aseptis binotata* in color and pattern. It is gray brown, has a full complement of dark lines and spots and a pale postreniform patch, and has a streaky hindwing with dark veins. The basal dash is thicker black than that of *Aseptis binotata* and extends fully to the antemedial line. Black wedges on the wing distal to the lower cell and in the fold are also more prominent in *Paraseptis adnixa*. In the Pacific Northwest, *Paraseptis adnixa* is typically patchy brown, often with a reddish tint, with a large pale ochre postreniform patch and contrasting black markings (Fig. 56). In most of California, it tends to be paler and less well marked with a speckled gray-brown or brown-gray forewing, less conspicuous postreniform patch and black marks, and slightly lighter hindwing. This form is even more similar to *Aseptis binotata* from the same region (Figs 53, 54). In the vicinity of Mono Lake in east-central California, *Paraseptis adnixa* is powdery pale gray with red-brown basal and postmedial areas, an off-white postreniform patch, and more dark streaks on the distal wing (Fig. 55).

Differences in genitalia between *Paraseptis* and *Aseptis* are described under the *Paraseptis* genus description. *Paraseptis adnixa* can usually be identified without dissection by the combination of notched hindwing, brown forewing with pale postreniform patch, and long basal dash.

**Figure 92.**
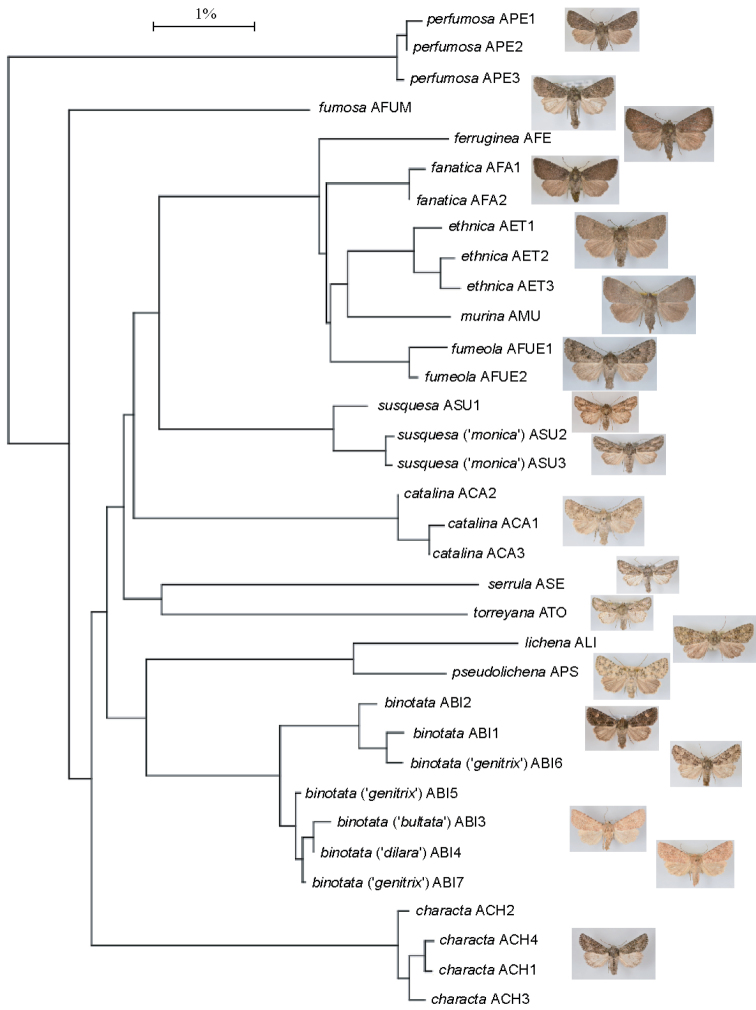
Neighbor-joining CO1 tree of *Aseptis*. The letter and number code after each species is the haplotype identifier as indicated in Table 1. An illustrative specimen for each species is shown on the right hand side. For *binotata* and *susquesa*, the geographical forms are also shown.

The CO1 barcodes of *Paraseptis* based on 59 samples from British Columbia to southern California demonstrate six major haplotype clusters separated by at least 0.5% (Fig. 92). Of these, three clusters separated by at least 1.8% (PAD4, PAD5, PAD6) are from California west of the Sierra Nevada divide. The other two are more divergent: two from southwestern British Columbia (PAD1+PAD3) and a single Washington specimen, and the other from near Mono Lake, California (PAD2). The BC/WA cluster differs by at least 2.6% from the Mono cluster and 2.5% from the CA cluster, and the Mono and CA clusters differ by at least 2.8%. Interestingly, the three most divergent clusters (BC/WA, Mono, and CA) correlate with the geographic variation described above. Although the alignment of barcode haplotypes and phenotypes could suggest the presence of more than one species, no consistent male or female genitalia differences were found to suggest the presence of more than a single species, and all male genitalia match those of the lectotype of *Hadena pausis* at USNM (slide #54). We therefore conclude that *Hadena pausis* Smith is a junior subjective synonym of *Hadena adnixa* Grote.

##### Distribution and biology.

This species is widely distributed along the Pacific Coast from northern Mexico to southwestern British Columbia. It is found mostly west of the divides of the Sierra Nevada, Cascades, and British Columbia Coast Mountains but there are at least three colonies east of these mountains: Inyo and Mono County, California; Klamath and Lake counties, Oregon; and interior British Columbia near Lillooet. *Paraseptis adnixa* is common in the Pacific Northwest, where it can be found in a variety of forested habitats. In southern California, it occurs in coastal chaparral and in oak and brush land in the foothills and mountains. The flight period is April to June in California and mid-May to August in the Pacific Northwest. The larva feeds on Indian plum (*Oemleria cerasiformis*) in the Rosaceae (Miller and Hammond 2000) and might be a specialist on it in parts of its range (including the Pacific Northwest). It has also been reported as feeding on Prunus (Rosaceae), which is the likely foodplant where the moth is found outside of the range of *Oemleria*, such as in interior British Columbia, south-central Oregon, and far-eastern California..

As described above, *Paraseptis adnixa* has three distinct populations based on superficial appearance and CO1 barcodes. Although there is little evidence to suggest more than a single species, we considered using subspecies to distinguish these forms. The Pacific Northwest populations are continuous to the border with California (Crabo et al. 2012) suggesting that a cline to the California form may exist in northern California. Until this is refuted it is best to consider these forms the ends of a north-south cline. By contrast, the eastern California populations near the border with Nevada are probably isolated. If distinguishing them with a subspecies epithet is desired the type locality of *Hadena adnixa* Grote should be restricted since the stated type locality, Nevada, could refer to Nevada or an unspecified site in eastern California (Lafontaine JD pers. comm. 2015).

#### Genus Viridiseptis gen. n.

Taxon classificationAnimaliaLepidopteraNoctuidae

http://zoobank.org/35396DDB-5784-4ACB-B5F9-821760B83BAF

##### Type species.

*Hadena marina* Grote, 1874.

##### Etymology.

The name is derived from *viridis* meaning green and *septis* by analogy to *Aseptis* and *Paraseptis*. The name is feminine.

##### Diagnosis.

*Viridiseptis* is a monotypic genus whose only member is found in California and adjacent Oregon. It is a stout small to medium-sized moth with a powdery mottled-green forewing.

The genitalia differ greatly from those of *Aseptis* and *Paraseptis*. In the male the distal half of the uncus is broad, flat, and covered densely by short fine hairs; the valve has triangular process from the inner surface of the sacculus, lacks an ampulla, has a thick blunt digitus, and the distal end is rhomboid without a typical cucullus or corona. The females of these genera are also highly divergent. That of *Viridiseptis* has soft, pad-like ovipositor lobes, very short apophyses, a sclerotized plate in the ventral wall of the proximal ductus bursae, and a membranous corpus bursae lacking signa with a membranous appendix bursae arising perpendicularly from the wall of the posterior corpus bursae rather than as a sclerotized posterior extension of it.

##### Description.

**Adult: Head**: Antenna filiform in both sexes. Frons smooth. Eye rounded, normal size, naked. Labial palpus unmodified, with moderately long second segment and short distal segment, reaching mid-eye. **Thorax**: Dorsal paired tufts on meso- and metathorax. Legs with tibiae lacking spiniform setae; tarsal segments with three rows of short spiniform setae on each segment. **Forewing**: Short and rounded, outer margin weakly scalloped, covered with olive-green, gray, black, and off-white scales, pattern of ordinary transverse lines, orbicular and reniform spots, but lacking distinct claviform spot and dashes. **Hindwing**: Venation typical of trifine noctuids with vein M2 weak, M2 closest to M1; outer margin contour weakly concave between veins M1 and M3, less prominent than in *Aseptis*. **Abdomen**: Base of male with paired hair-pencils, complete with levers and pockets. Weak dorsal scale tufts on proximal segments. **Male genitalia** (Fig. 75): Tegumen shape unmodified without narrower area near uncus; penicillus broad, rounded. Uncus base, narrow, cylindrical, distal two-thirds thicker and wider, roughly canoe shaped with proximal and distal tapered areas separated by even-width segment, dorsal distal portion and undersurface of tip covered densely with short hairs. Saccus of vinculum long and narrow. Juxta base broadly shield shaped, tapering to slight waist at junction with apical third, apical segment at base of aedeagus expanded to slightly wider than “waist” with raised sclerotized structure with slightly overhanging lateral edges and rounded tip. Valve narrow, 6× as long as narrow mesial section at end of sacculus, slightly curved dorsally; sacculus 0.4× as long as valve, reaching 2/3× to base of costa, with tooth-like triangular process on distal portion near ventral part of clasper; cucullus large, costal portion thick and apex and ventral portions thin, rhomboid with three points: right-angle point at dorsal base, slightly acute apex lacking a corona, and more rounded and obtuse ventral margin; clasper reduced to attachment on valve, ampulla absent; digitus arising at ventral cucullus from weak plate on ventral distal valve, stout, short, tooth-like or curved ventrad. Aedeagus 5× as long as wide, distal third curved slightly ventrad, with abrupt reduction in caliber at mid-point from bull-nosed sclerotized ridge across ventral wall, a patch of distal striae with long extensions onto vesica and patch of small spines near ventral apex; vesica slightly shorter than aedeagus, bent nearly 90° ventrad and to right at base, then curved slightly leftward to end with tip ventrad to end of aedeagus, with moderate-sized basally-constricted diverticulum on posterior wall at 1/3 from base and smaller dome-shaped diverticulum on left wall at 2/3 from base, cornuti absent. **Female genitalia** (Fig. 91): Papilla analis broadly triangular with rounded tip, covered by hair-like posteriorly-directed setae that are shorter and more dense at tip; segment VIII and apophyses very short; median posterior 7^th^ sternite concave at ostium bursae; ostium bursae broad, weakly sclerotized; ductus bursae cylindrical, 4× length of segment VIII, membranous with granulose sclerotized plate with thicker posterior portion forming a slight lip in ventral wall ¼ distance from ostium to corpus bursae; corpus bursae membranous without signa, pear shaped with narrow posterior and larger ovoid anterior portions, ~1.7× as long as ductus bursae; appendix bursae extending ventrad and slightly rightward perpendicular to corpus bursae from origin on posterior ventral wall of corpus bursae, membranous, ovoid, ~1/3 size of corpus bursae, with junction with ductus seminalis at left posterior base near corpus bursae.

##### Discussion.

The higher classification of this genus is enigmatic. Although *Viridiseptis* clusters with genera in the tribe Xylenini by CO1 barcodes and its hindwing notch suggests an affinity to *Aseptis* and *Paraseptis*, absence other structural similarities between them and the biology of its larva suggest that the recent association with *Aseptis* is incorrect. The distal male valve of *Viridiseptis* bears some resemblance to other genera in the Xylenini such as *Sunira* Franclemont, but the valve differs greatly in other respects such as absence of the ampulla. A long twisted ampulla is one of the defining characters of the subtribe Xylenina (Fibiger and Lafontaine 2005). In addition, the larva of *Viridiseptis* feeds externally on forbs rather than woody plants, a defining character of the entire tribe Xylenini (op. cit.), further clouding the relationship. There is little evidence that *Viridiseptis* is related closely to *Oligia* Hübner where it had been placed (Franclemont and Todd 1983) prior to the most recent check list (Lafontaine and Schmidt 2010). For these reasons, the phylogenetic position of *Viridiseptis* in the Noctuinae is uncertain. We suggest that it be placed in the Xylenini (incertae sedis) section pending a more encompassing revision of the subfamily.

The ridge on the ventral male aedeagus and plate in the ventral wall of the female ductus bursae might be engaged during copulation.

#### Viridiseptis marina

Taxon classificationAnimaliaLepidopteraNoctuidae

(Grote, 1874), comb n.

Figs 57 , 58 , 75 , 91

Hadena marina Grote, 1874a: 67.

##### Type material.

**Holotype** female [BMNH, photograph examined]. Type locality: California.

##### Diagnosis.

A medium-sized or slightly smaller than average stout species, wingspan 31.9±1.5 mm (n = 25; range 29.5–35 mm). The forewing is granular mossy green, occasionally yellowish green, with mottled dark-gray to black and light-green pattern that obscures all but the darkest parts of the lines and spots. The darkest areas are a small patch at the base of the trailing edge of the wing, the cell and fold in the medial area, a bar on the costa preceding the subterminal line, and terminal area opposite the reniform spot. The relatively small reniform spot and round orbicular spot are filled with peripheral whitish and central green scales. The basal, antemedial and postmedial lines are double, black filled with green, and the subterminal line is green; all are sinuous and appear incomplete. The fringe is checkered green and gray. The hindwing is light brown gray with a darker border.

This species can usually be identified by superficial appearance alone. If in doubt, the male genitalia described under the genus description are diagnostic.

##### Distribution and biology.

*Viridiseptis marina* occurs throughout coastal California and in southwestern Oregon as far north as Douglas County. It is widely distributed in southern California, where it is often common. It is found in many habitats such as coastal chaparral, mountain forest, mountain-desert transition zone, and occasionally in the deserts from sea level to at least 2000 m. It flies from April to early July. The larva feeds on forbs and has been recorded on *Nemophila* spp. and *Pholistoma auritium* (Lindl.) Lilja (Hydrophyllaceae) and *Lithophragma* spp. (Saxifragaceae) (Robinson et al. 2010).

## Discussion

This revision should help clear up confusion in the genus *Aseptis* by facilitating identification of the species—in no small part impeded prior to this study by more names than actual species—and by refining the generic relationships of species included with *Aseptis* in recent check lists. Of the genera treated herein, *Aseptis* and *Paraseptis* are retained in the tribe Xylenini, subtribe Xylenina and *Viridiseptis* is transferred to Xylenini (incertae sedis).

**Figure 93.**
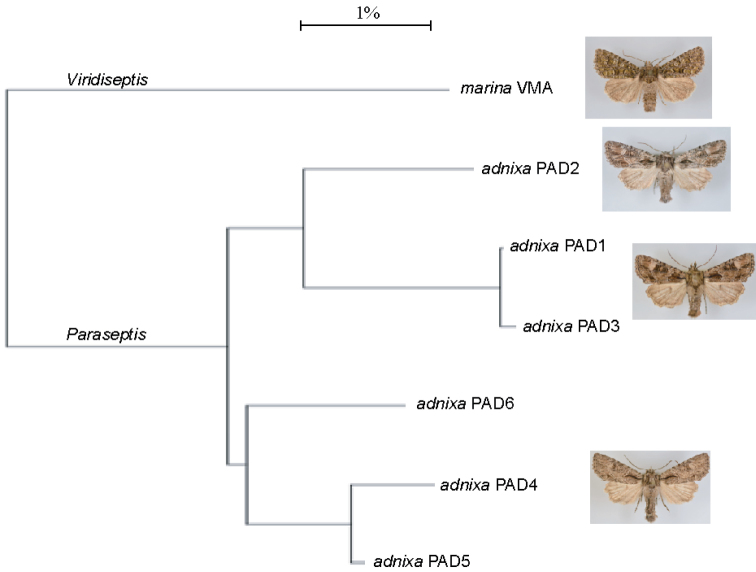
Neighbor-joining CO1 tree of *Paraseptis* and *Viridiseptis*. The letter and number code after each species is the haplotype identifier as indicated in Table 1. Illustrative specimens for *Viridiseptis marina* and the three geographical phenotypes of *adnixa* are shown on the right hand side.

Although our work is based primarily on structural evidence, we also accessed a large pre-existing data base of CO1 barcodes available at BOLD and tested our theories against it during the course of this work. We observed, for the most part, a good correlation between structural and molecular data. Lack of significant variation in two variable widespread species, *Aseptis binotata* and *Aseptis characta*, supported pre-existing synonomies in both species and the proposed new ones in *Aseptis binotata*, and was reassuring evidence that no cryptic species were being overlooked. As recently reported for a number of Canadian moths (Zahiri et al. 2014), we found additional instances of morphologically and biologically well-defined species with no more than 1.5% difference in CO1 DNA sequence, e.g., within the *Aseptis fumeola* species group (Fig. 92), as well as examples of structurally well-delineated species within which there is greater diversity of CO1 DNA. The latter was most evident in *Paraseptis adnixa* which has several major barcode haplotype clusters that differ by up to 2.8% (Fig. 93).

## Supplementary Material

XML Treatment for Aseptis

XML Treatment for Aseptis fumeola

XML Treatment for Aseptis ethnica

XML Treatment for Aseptis murina

XML Treatment for Aseptis ferruginea

XML Treatment for Aseptis fanatica

XML Treatment for Aseptis lichena

XML Treatment for Aseptis pseudolichena

XML Treatment for Aseptis binotata

XML Treatment for Aseptis catalina

XML Treatment for Aseptis serrula

XML Treatment for Aseptis torreyana

XML Treatment for Aseptis susquesa

XML Treatment for Aseptis fumosa

XML Treatment for Aseptis perfumosa

XML Treatment for Aseptis characta

XML Treatment for Paraseptis

XML Treatment for Paraseptis adnixa

XML Treatment for Viridiseptis

XML Treatment for Viridiseptis marina

## Check list of the species of *Aseptis* McDunnough, *Paraseptis* Mustelin & Crabo, and *Viridiseptis* Mustelin & Crabo

*Aseptis* McDunnough, 1937

*fumeola* (Hampson, 1918)

= *probata* (Barnes & McDunnough, 1910)

*ethnica* (Smith, 1899)

*murina* Mustelin, 2000

*ferruginea* Mustelin, 2000

*fanatica* Mustelin, 2006

*lichena* (Barnes & McDunnough, 1912)

*pseudolichena* Mustelin & Leuschner, 2000

*binotata* (Walker, 1865)

= *rubiginosa* (Walker, 1865)

= *extersa* (Walker, 1865)

= *paviae* (Strecker, 1874)

= *curvata* (Grote, 1874)

= *genitrix* (Grote, 1878)

= *inconspicua* (Smith, 1893), **nomen nudum**

= *dilara* (Strecker, 1899)

= *bultata* (Smith, 1906)

= *cara* (Barnes & McDunnough, 1918)

*catalina* (Smith, 1899)

*serrula* (Barnes & McDunnough, 1918)

*torreyana* Mustelin, 2006

*susquesa* (Smith, 1908)

= *monica* (Barnes & McDunnough, 1918)

*fumosa* (Grote, 1879)

*perfumosa* (Hampson, 1918)

*characta* (Grote, 1880)

= *erica* (Smith, 1905)

= *luteocinerea* (Smith, 1900)

= *pluraloides* (McDunnough, 1922)

*Paraseptis* Mustelin & Crabo, 2015

*adnixa* (Grote, 1880)

= *pausis* (Smith, 1899)

*Viridiseptis* Mustelin & Crabo, 2015

*marina* (Grote, 1874)
